# Albumin, urea‐to‐albumin ratio, or the albumin‐to‐creatinine ratio to predict outcomes in heart failure with mildly reduced ejection fraction

**DOI:** 10.1111/eci.70165

**Published:** 2025-12-26

**Authors:** Alexander Schmitt, Ibrahim Akin, Marielen Reinhardt, Noah Abel, Felix Lau, Jonas Dudda, Mohammad Abumayyaleh, Kathrin Weidner, Thomas Bertsch, Daniel Duerschmied, Michael Behnes, Tobias Schupp

**Affiliations:** ^1^ Department of Cardiology, Angiology, Haemostaseology and Medical Intensive Care, Medical Faculty Mannheim University Medical Centre Mannheim, Heidelberg University Heidelberg Germany; ^2^ Institute of Clinical Chemistry, Laboratory Medicine and Transfusion Medicine, Nuremberg General Hospital Paracelsus Medical University Nuremberg Germany

**Keywords:** albumin, albumin‐derived ratios, heart failure with mildly reduced ejection fraction, HFmrEF, prognosis

## Abstract

**Background:**

This study investigates the prognostic impact of albumin, the urea‐to‐albumin ratio (UAR), and albumin‐to‐creatinine ratio (ACR) in patients with heart failure with mildly reduced ejection fraction (HFmrEF), since hypoalbuminemia, renal disease and malnutrition often coincide with heart failure (HF).

**Methods:**

Consecutive patients hospitalized with HFmrEF at one university medical centre were retrospectively included from 2016 to 2022. Patients were stratified into quartiles based on albumin, the UAR, and ACR. The primary endpoint was all‐cause mortality at 30 months (median follow‐up), key secondary endpoint was long‐term HF‐related rehospitalization.

**Results:**

The study cohort comprised 2,061 patients with HFmrEF with a median albumin level of 32.4 g/L. Albumin levels, the UAR and ACR were predictive for the risk of long‐term all‐cause mortality, which was still observed after multivariable adjustment (albumin Q1 vs. Q4: HR = 2.260; 95% CI 1.623–3.148; *p* = .001 / UAR Q4 vs. Q1: HR = 1.507; 95% CI 1.071–2.119; *p* = .019/ACR Q1 vs. Q4: HR = 2.208; 95% CI 1.528–3.190; *p* = .001). However, neither albumin nor the UAR or ACR predicted the risk of HF‐related rehospitalization (albumin Q1 vs. Q4: HR = 1.117; 95% CI .678–1.842; *p* = .664 / UAR Q4 vs. Q1: HR = 1.589; 95% CI .922–2.738; *p* = .095 / ACR Q1 vs. Q4: HR = 1.112; 95% CI .624–1.981; *p* = .720).

**Conclusions:**

Hypoalbuminemia is common in hospitalized HFmrEF patients. Low albumin levels, ACRs, and elevated UARs independently predicted long‐term all‐cause mortality, but not HF‐related rehospitalization. The UAR and ACR did not provide a clinically significant predictive advantage over albumin levels alone.

**Trial Registration:**

ClinicalTrials.gov Identifier: NCT05603390 (date of registration: 10.10.2020)

## Graphical abstract

Among 2061 hospitalized HFmrEF patients, low albumin, albumin‐to‐creatinine ratios (ACR), and elevated urea‐to‐albumin ratios (UAR) independently predicted long‐term all‐cause mortality, but not HF‐related rehospitalization. The UAR and ACR did not provide a clinically significant predictive advantage over albumin levels alone.

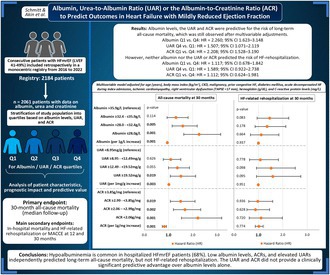

## 1 BACKGROUND

Heart failure (HF) affects over 64 million people worldwide, with its prevalence and healthcare burden expected to rise due to demographic changes.^1^, ^2^ However, despite advancements in predicting and improving patients' prognosis over previous decades, the decline in mortality has plateaued since the early 21st century and the prognostication in HF patients remains challenging, requiring a more granular approach across distinct HF phenotypes.^3^, ^4^ In response, recent European HF guidelines have emphasized the need for further research on prognostic biomarkers, particularly in patient groups previously underrepresented in clinical studies. Consequently, HF with mildly reduced ejection fraction (HFmrEF) was recently introduced as a distinct category to bridge the traditional dichotomy between HF with reduced or preserved ejection fraction (HFrEF and HFpEF) and to expand the limited body of evidence guiding clinical management of these patients.^5^, ^6^ HFmrEF represents a heterogeneous entity, encompassing patients with features overlapping HFrEF or HFpEF, underscoring the need for targeted prognostic data to determine whether widely available biomarkers provide prognostic value within this intermediate phenotype.^7^

In recent decades, biomarkers such as amino‐terminal prohormone of brain natriuretic peptide (NT‐proBNP) and cardiac troponins have emerged as valuable tools for risk stratification and prognostication in HF.^8^, ^9^, ^10^ However, the utility of alternative biomarkers reflecting renal function and the nutritional and inflammatory status of patients, such as albumin, the urea‐to‐albumin ratio (UAR) or the albumin‐to‐creatinine ratio (ACR), have not been thoroughly explored, especially in patients with HFmrEF. Even though observational studies have demonstrated that chronic kidney disease (CKD) and hypoalbuminemia are common in HF patients, with prevalences reaching 48% and 54%, respectively.^11^, ^12^, ^13^

Albumin, a negative acute‐phase protein synthesized by the liver, serves as a marker of both nutritional status, as well as systemic inflammation and plays a crucial role in maintaining colloid osmotic pressure. Hypoalbuminemia has been associated with adverse outcomes in various cardiovascular conditions, likely attributable to its association with chronic inflammation, malnutrition, and fluid overload.^14^, ^15^, ^16^ However, its predictive value in HFmrEF remains unclear. While urea is sensitive to acute metabolic changes and reflects catabolic states, dehydration and protein turnover, creatinine is more stable and resembles a reliable indicator of renal function and muscle mass.^17^, ^18^ By integrating these factors, the UAR and ACR may better capture the burden of comorbidity and systemic alterations seen in HF patients.

Therefore, this study aims to evaluate and compare the predictive value of albumin levels, the UAR, and the ACR in a large‐scale retrospective registry‐based analysis of consecutive patients hospitalized with HFmrEF.

## 2 METHODS

### 2.1 Study patients, design and data collection

For the present study, all consecutive patients hospitalized with HFmrEF at the University Medical Centre Mannheim were included from January 2016 to December 2022, as recently published.^19^ The present study was derived from the ‘Heart Failure With Mildly Reduced Ejection Fraction Registry’ (HARMER), representing a retrospective single‐centre all‐comers registry including consecutive patients with HFmrEF hospitalized at the University Medical Centre Mannheim, Germany (clinicaltrials.gov Identifier: NCT05603390). The registry was conducted according to the principles of the Declaration of Helsinki and was approved by the Medical Ethics Committee II of the Medical Faculty Mannheim, University of Heidelberg, Germany (ethical approval code: 2022‐818).

### 2.2 Inclusion and exclusion criteria

All consecutive patients ≥18 years of age hospitalized with HFmrEF at one institution were included. All included patients underwent at least one standardized transthoracic echocardiography at the cardiologic department during the index hospitalization, where the diagnosis of HFmrEF was assessed. The diagnosis of HFmrEF was determined retrospectively according to the ‘2021 European Society of Cardiology (ESC) guidelines for the diagnosis and treatment of acute and chronic HF’.^6^ Accordingly, all patients with a left ventricular ejection fraction (LVEF) of 41%–49% and symptoms and/or signs of HF were included. Elevated NT‐proBNP levels and other evidence of structural heart disease supported the diagnosis but were not mandatory for the diagnosis of HFmrEF. Standardized transthoracic echocardiography was performed by cardiologists during routine clinical care in accordance with current international guidelines.^20^, ^21^ Transthoracic echocardiography was performed using the Vivid™ 7, Vivid E9 and Vivid E95 ultrasound systems with EchoPac™ software (all from GE HealthCare, Chicago, IL, USA). For the present study, patients without a measurement of albumin, urea, and/or creatinine levels during the index hospitalization were excluded. No further exclusion criteria were applied.

### 2.3 Risk stratification

Risk stratification was performed according to albumin levels, the UAR and the ACR during the index hospitalization. In patients with multiple albumin, urea or creatinine measurements, the laboratory value closest to the date of the index echocardiography was selected for analysis (i.e. diagnosis of HFmrEF). Albumin (g/L), urea (mg/dL), and creatinine levels (mg/dL) were measured within serum or plasma, which was tolerated since deviations between these blood‐derived samples are clinically insignificant. Albumin was measured using the *Atellica® CH Albumin BCP (AlbP)* assay (Siemens Healthineers, Erlangen, Germany), which is based on the bromocresol purple colorimetric method according to Carter and Louderback. The endpoint reaction is measured at 596/694 nm. Urea was determined using the *Atellica® CH Urea Nitrogen* assay (Siemens Healthineers, Erlangen, Germany). The method follows the enzymatic procedure described by Roch and Ramel, involving urease and glutamate dehydrogenase. Results expressed as blood urea nitrogen (BUN) were converted to urea using the formula: BUN × 2.143 = urea. Creatinine was quantified using the *Atellica® CH Creatinine_2* assay (Siemens Healthineers, Erlangen, Germany), which is based on the Jaffe method (reaction of picric acid with creatinine in alkaline solution). The end product is measured photometrically at 505/571 nm.

Patients were stratified into quartiles depending on the distribution of albumin levels, the UAR and ACR. Calculation of the corresponding quartiles for albumin levels resulted in the following stratification: Q1 = albumin ≤28 g/L, Q2 = albumin >28–<32.4 g/L, Q3 = albumin ≥32.4–≤35.9 g/L, and Q4 = albumin >35.9 g/L. Calculation of the corresponding quartiles for the UAR resulted in the following stratification: Q1 = UAR <8.95 mg/g, Q2 = UAR ≥8.95–<12.49 mg/g, Q3 = UAR ≥12.49–<19.52 mg/g, and Q4 = UAR ≥19.52 mg/g. Calculation of the corresponding quartiles for the ACR resulted in the following stratification: Q1 = ACR <2.06 g/mg, Q2 = ACR ≥2.06–<2.99 g/mg, Q3 = ACR ≥2.99–<3.85 g/mg, and Q4 = ACR ≥3.85 g/mg. In addition, the prognostic impact of albumin levels, the UAR and ACR was investigated as continuous variables within multivariable Cox regression analyses.

### 2.4 Study endpoints

The primary endpoint was long‐term all‐cause mortality (i.e. at the median follow‐up of 30 months). Secondary endpoints included in‐hospital mortality, all‐cause mortality, HF‐related rehospitalization, and major adverse cardiac and cerebrovascular events (MACCE) at 12 months, as well as HF‐related rehospitalization, cardiac rehospitalization, acute myocardial infarction (AMI), stroke, coronary revascularization, and MACCE at long‐term follow‐up. All‐cause mortality was documented using the electronic hospital information system and by directly contacting state resident registration offices (‘Bureau of Mortality Statistics’). HF‐related hospitalization was defined as rehospitalization due to worsening HF requiring intravenous diuretic therapy. HF‐related rehospitalization comprised patients with hospitalization due to worsening HF as the primary cause, or as a result of another cause but associated with worsening HF at the time of admission, or as a result of another cause but complicated by worsening HF during its course. Moreover, prior congestive HF was defined as a previous diagnosis of HF prior to the index admission documented in the hospital information system together with signs and or symptoms indicative of congestion (peripheral oedema, dyspnoea, pulmonary congestion, etc.). Cardiac rehospitalization was defined as rehospitalization due to a primary cardiac condition, including worsening HF, AMI, coronary revascularization, and symptomatic atrial or ventricular arrhythmias. MACCEs were defined as the composite of all‐cause mortality, coronary revascularization, non‐fatal AMI and non‐fatal stroke.

### 2.5 Statistical methods

Quantitative data were presented as median with interquartile ranges (IQRs). They were compared using the Kruskal‐Wallis test. Deviations from a Gaussian distribution were tested by the Kolmogorov–Smirnov test. Qualitative data were presented as absolute and relative frequencies and were compared using the chi‐square test or the Fisher's exact test, as appropriate. Spearman's rank correlation for nonparametric data was used to test for the association of albumin levels, the UAR, and the ACR with baseline characteristics as well as medical and laboratory parameters obtained during the index admission.

Kaplan–Meier analyses were performed stratified by the calculated albumin, UAR or ACR quartiles and univariable hazard ratios (HRs) were given together with 95% confidence intervals (CIs). The prognostic impact of albumin, the UAR and the ACR was then investigated within a multivariable Cox regression model adjusted for age (years), body mass index (BMI, kg/m^2^), CKD, malignancy, prior congestive HF, diabetes mellitus, ADHF during the index admission, ischemic cardiomyopathy, right ventricular dysfunction (defined as a tricuspid annular plane systolic excursion <17 mm), hemoglobin (g/dL), and C‐reactive protein levels (mg/L). Furthermore, the same multivariable Cox regression model was applied within specific subgroups stratified by estimated glomerular filtration rate (eGFR, calculated based on the CKD‐EPI equation^22^), the presence of diabetes mellitus and prior congestive HF. Covariates for the multivariable model were selected based on clinical relevance and results from univariable analyses identifying variables associated with the primary endpoint (*p* ≤ .1). The corresponding univariable results are provided in Table S5. Post‐hoc power calculations were conducted for the multivariable Cox regression model. The effective number of events was estimated by accounting for the proportion of outcome variance explained by covariates in each model. Using this information, the minimum detectable hazard ratios for the Q1 versus Q4 comparisons of albumin, UAR and ACR were calculated at power levels of 80% and 90% according to the method described by Schoenfeld.^23^

Results of all statistical tests were considered significant for *p* ≤ 0.05. SPSS (Version 25, IBM, Armonk, NY, USA) was used for all statistical analyses.

## 3 RESULTS

### 3.1 Study population

**FIGURE 1:**
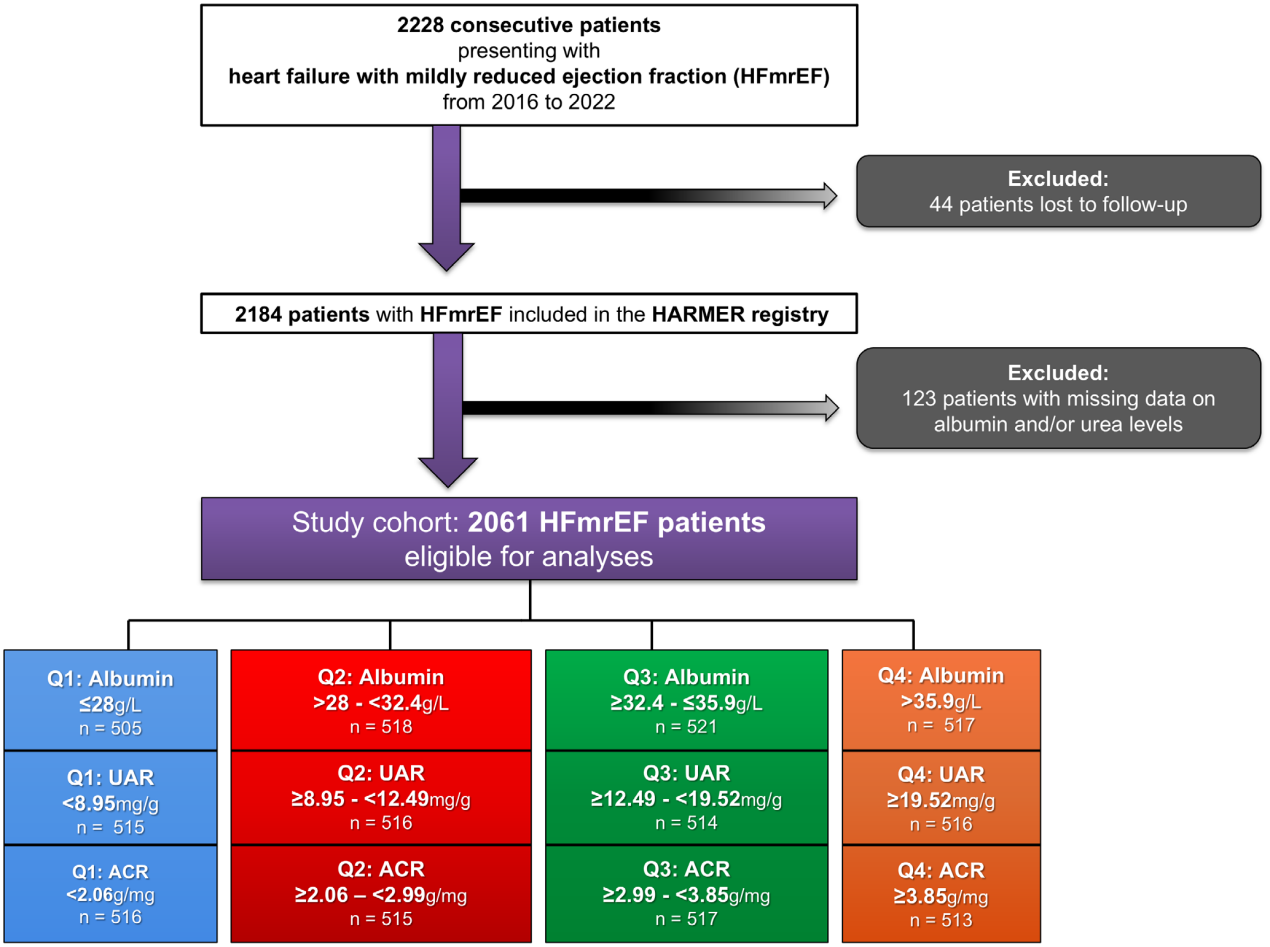
Flowchart of the study cohort and stratification criteria. ACR, albumin‐to‐creatinine ratio; HARMER, Heart Failure with Mildly Reduced Ejection Fraction Registry; UAR, urea‐to‐albumin ratio.

2228 consecutive patients with HFmrEF were included in the HARMER registry from 2016 to 2022. The lost to follow‐up rate regarding all‐cause mortality was 1.97% (*n* = 44) resulting in a registry cohort of 2184 HFmrEF patients. For the present study, 123 patients were excluded due to missing data on albumin, urea and/or creatinine, yielding a final study cohort of 2061 patients (Figure 1; flowchart). Median albumin, urea and creatinine levels within the entire study cohort were 32.4 g/L (IQR 28.2–36.0), 40.2 mg/dL (IQR 29.9–57.8) and 1.07 mg/dL (IQR 0.86–1.45), respectively. In total, 67.9% of patients (*n* = 1400) were considered to have hypoalbuminemia defined as an albumin level < 35 g/L.

As displayed in Table 1, stratification by albumin quartiles demonstrated that HFmrEF patients with lower albumin levels were older and had lower median BMI. Furthermore, CKD and malignancies were more prevalent in patients with lower albumin levels, while rates for arterial hypertension and diabetes mellitus did not significantly differ across the analysed subgroups. Regarding comorbidities during the index hospitalization, patients with lower albumin levels suffered more frequently from ADHF, as well as cardiogenic shock. The use of cardiovascular pharmacotherapies on admission was similar across albumin quartiles, except for loop diuretics, which were more frequently prescribed in patients with lower albumin levels (Table 1).

**TABLE 1:** Baseline characteristics stratified by albumin quartiles.

	Q1: Albumin ≤28g/L (*n* = 505)		Q2: Albumin >28 ‐ <32.4g/L (*n* = 518)		Q3: Albumin ≥32.4–≤35.9g/L (*n* = 521)		Q4: Albumin >35.9g/L (*n* = 517)		*p*
Age, median (IQR)	77	(69‐83)	77	(65‐84)	75	(64–83)	71	(57‐80)	.**001**
Male sex, *n* (%)	297	(58.8)	327	(63.1)	336	(64.5)	371	(71.8)	.**001**
Body mass index, kg/m^2^, median (IQR)	25.4	(22.8‐29.1)	25.9	(23.2‐30.1)	26.7	(24.2–30.9)	27.7	(24.6‐31.2)	.**001**
SBP, mmHg, median (IQR)	135	(115‐156)	140	(123‐163)	146	(130–167)	146	(132‐167)	.**001**
DBP, mmHg, median (IQR)	72	(61‐84)	78	(69‐89)	80	(70–94)	81	(72‐93)	.**001**
Heart rate, bpm, median (IQR)	84	(70‐99)	80	(68‐95)	80	(69–93)	79	(67‐94)	.**014**
Medical history, *n* (%)									
Coronary artery disease	202	(40.0)	205	(39.6)	213	(40.9)	219	(42.4)	.808
Prior myocardial infarction	103	(20.4)	128	(24.7)	132	(25.3)	122	(23.6)	.251
Prior PCI	123	(24.4)	146	(28.2)	141	(27.1)	160	(30.9)	.126
Prior CABG	52	(10.3)	48	(9.3)	54	(10.4)	49	(9.5)	.908
Prior valvular surgery	17	(3.4)	26	(5.0)	20	(3.8)	28	(5.4)	.334
Congestive heart failure	178	(35.2)	176	(34.0)	169	(32.4)	165	(31.9)	.663
Prior LVEF available, *n* (%)	167	(33.1)	193	(37.3)	181	(34.7)	196	(37.9)	.337
Prior LVEF, median (IQR)	51 (43–60)		50 (44–60)		50 (45–60)		50 (42–60)		.892
Prior LVEF ≥50%	95	(56.9)	111	(57.5)	99	(54.7)	106	(54.1)	.889
Prior LVEF 41‐49%	34	(20.4)	39	(20.2)	44	(24.3)	43	(21.9)	.762
Prior LVEF ≤40%	21	(12.6)	19	(9.8)	18	(9.9)	17	(8.7)	.666
Decompensated heart failure within 12 months prior to index	60	(11.9)	62	(12.0)	57	(10.9)	38	(7.4)	.051
Prior ICD	7	(1.4)	9	(1.7)	8	(1.5)	16	(3.1)	.172
Prior sICD	1	(0.2)	3	(0.6)	2	(0.4)	2	(0.4)	.811
Prior CRT‐D	7	(1.4)	8	(1.5)	6	(1.2)	8	(1.5)	.942
Prior Pacemaker	45	(8.9)	47	(9.1)	46	(8.8)	50	(9.7)	.965
Chronic kidney disease	219	(43.4)	177	(34.2)	138	(26.5)	114	(22.1)	.**001**
Peripheral artery disease	73	(14.5)	64	(12.4)	51	(9.8)	42	(8.1)	.**007**
Stroke	67	(13.3)	85	(16.4)	86	(16.5)	80	(15.5)	.448
Liver cirrhosis	24	(4.8)	10	(1.9)	4	(0.8)	5	(1.0)	.**001**
Malignancy	130	(25.7)	75	(14.5)	59	(11.3)	47	(9.1)	.**001**
COPD	80	(15.8)	65	(12.5)	56	(10.7)	46	(8.9)	.**005**
Cardiovascular risk factors, *n* (%)									
Arterial hypertension	398	(78.8)	397	(76.6)	404	(77.5)	409	(79.1)	.756
Diabetes mellitus	203	(40.2)	198	(38.2)	189	(36.3)	168	(32.5)	.068
Hyperlipidemia	125	(24.8)	149	(28.8)	157	(30.1)	196	(37.9)	.**001**
Smoking	162	(32.1)	186	(35.9)	188	(36.1)	222	(42.9)	.**004**
Current	77	(15.2)	88	(17.0)	108	(20.7)	118	(22.8)	.**008**
Former	85	(16.8)	98	(18.9)	80	(15.4)	104	(20.1)	.188
Family history	20	(4.0)	40	(7.7)	56	(10.7)	73	(14.1)	.**001**
Comorbidities at index hospitalization, *n* (%)									
Acute coronary syndrome									
Unstable angina	6	(1.2)	18	(3.5)	29	(5.6)	43	(8.3)	.**001**
STEMI	30	(5.9)	56	(10.8)	54	(10.4)	29	(5.6)	.**001**
NSTEMI	78	(15.4)	72	(13.9)	65	(12.5)	52	(10.1)	.068
Acute decompensated heart failure	175	(34.7)	127	(24.5)	99	(19.0)	65	(12.6)	.**001**
Cardiogenic shock	29	(5.7)	15	(2.9)	7	(1.3)	1	(.2)	.**001**
Atrial fibrillation	232	(45.9)	223	(43.1)	207	(39.7)	197	(38.1)	.053
Cardiopulmonary resuscitation	23	(4.6)	19	(3.7)	6	(1.2)	3	(.6)	.**001**
Out‐of‐hospital	8	(1.6)	8	(1.5)	4	(.8)	2	(.4)	.164
In‐hospital	15	(3.0)	11	(2.1)	2	(.4)	1	(.2)	.**001**
Stroke	54	(10.7)	88	(17.0)	69	(13.2)	87	(16.8)	.**010**
Medication on admission, *n* (%)									
ACE‐inhibitor	172	(34.1)	185	(35.7)	186	(35.7)	195	(37.7)	.683
ARB	100	(19.8)	107	(20.7)	128	(24.6)	120	(23.2)	.222
Beta‐blocker	302	(59.8)	287	(55.4)	287	(55.1)	287	(55.5)	.374
Aldosterone antagonist	45	(8.9)	44	(8.5)	46	(8.8)	50	(9.7)	.926
ARNI	3	(0.6)	5	(1.0)	3	(0.6)	5	(1.0)	.808
SGLT2‐inhibitor	5	(1.0)	15	(2.9)	9	(1.7)	13	(2.5)	.137
Loop diuretics	237	(46.9)	204	(39.4)	179	(34.4)	149	(28.8)	.**001**
Statin	214	(42.4)	229	(44.2)	237	(45.5)	251	(48.5)	.242
ASA	174	(34.5)	163	(31.5)	183	(35.1)	174	(33.7)	.624
P2Y12‐inhibitor	45	(8.9)	48	(9.3)	47	(9.0)	54	(10.4)	.825
DOAC	109	(21.6)	131	(25.3)	122	(23.4)	122	(23.6)	.581
Vitamin K antagonist	36	(7.1)	53	(10.2)	44	(8.4)	44	(8.5)	.366

*Note*: Level of significance *p* ≤ .05. Bold type indicates statistical significance.

Abbreviations: ACE, angiotensin‐converting‐enzyme; ARB, angiotensin receptor blocker; ARNI, angiotensin receptor neprilysin inhibitor; ASA, acetylsalicylic acid; CABG, coronary artery bypass grafting; CKD, chronic kidney disease; COPD, chronic obstructive pulmonary disease; CRT‐D, cardiac resynchronization therapy with defibrillator; DBP, diastolic blood pressure; DOAC, directly acting oral anticoagulant; IQR, interquartile range; PCI, percutaneous coronary intervention; (N)STEMI, non‐ST‐segment elevation myocardial infarction; SBP, systolic blood pressure; SGLT2, sodium glucose linked transporter 2; (s) ICD, (subcutaneous) implantable cardioverter defibrillator.

HF‐related and procedural data (Table 2) revealed no differences in the primary underlying HF etiologies between the albumin quartiles except for a higher prevalence of hypertensive cardiomyopathy in the albumin quartiles Q3 and Q4. However, heart valve diseases (i.e. aortic valve stenosis and mitral or tricuspid regurgitation) were more prevalent in patients with lower albumin levels (*p* ≤ .030 for all three comparisons). While the distribution of coronary artery disease and ischemic cardiomyopathy was similar across albumin quartiles, patients in Q1 underwent coronary angiography less often than those with higher albumin levels. Regarding baseline laboratory data, patients with lower albumin levels presented with worse renal function, lower iron and transferrin levels, low‐density lipoprotein (LDL) and high‐density lipoprotein (HDL) cholesterol as well as higher NT‐proBNP levels. Furthermore, hemoglobin, C‐reactive protein (CRP) levels, and white blood cell counts were elevated in patients with lower albumin levels (*p* ≤ .001 for all mentioned comparisons). Finally, prescription of pharmacotherapies at discharge demonstrated lower rates of angiotensin receptor blocker, sodium‐glucose cotransporter‐2 inhibitor, and statin use in patients with lower albumin levels, whereas loop diuretics were more commonly prescribed in these patients (*p* ≤ .033 for all comparisons) (Table 2).

**TABLE 2:** Heart failure‐related and procedural data.

	Q1: Albumin ≤28g/L (*n* = 505)		Q2: Albumin >28–<32.4g/L (*n* = 518)		Q3: Albumin ≥32.4–≤35.9g/L (*n* = 521)		Q4: Albumin >35.9g/L (*n* = 517)		*p*
Heart failure etiology, *n* (%)									
Ischemic cardiomyopathy	279	(55.2)	311	(60.0)	314	(60.3)	287	(55.5)	.185
Non‐ischemic cardiomyopathy	39	(7.7)	39	(7.5)	26	(5.0)	43	(8.3)	.165
Hypertensive cardiomyopathy	31	(6.1)	30	(5.8)	53	(10.2)	50	(9.7)	.**011**
Congenital heart disease	0	(0.0)	1	(0.2)	1	(0.2)	2	(0.4)	.578
Valvular heart disease	23	(4.6)	29	(5.6)	22	(4.2)	19	(3.7)	.501
Tachycardia associated	27	(5.3)	22	(4.2)	37	(7.1)	34	(6.6)	.200
Tachymyopathy	6	(1.2)	7	(1.4)	7	(1.3)	16	(3.1)	.061
Pacemaker‐induced cardiomyopathy	5	(1.0)	6	(1.2)	4	(0.8)	4	(.8)	.896
Unknown	101	(20.0)	80	(15.4)	64	(12.3)	78	(15.1)	.**008**
NYHA functional class, *n* (%)									
I/II	328	(64.9)	369	(71.3)	388	(74.4)	414	(80.1)	.**001**
III	116	(23.0)	97	(18.7)	92	(17.7)	80	(15.5)	
IV	61	(12.1)	52	(10.0)	41	(7.9)	23	(4.4)	
Echocardiographic data									
LVEF, %, median (IQR)	45 (45–46)		45 (45–47)		45 (45–47)		45 (45–47)		.314
IVSd, median (IQR)	11 (10–13)		12 (11–13)		12 (11–13)		12 (11–13)		.**028**
LVEDD, mm, median (IQR)	48 (44–54)		49 (44–54)		49 (44–53)		49 (45–54)		.444
TAPSE, mm, median (IQR)	20 (17–23)		20 (17–23)		20 (17–23)		20 (18–23)		.**037**
LA diameter, mm, median (IQR)	42 (37–47)		42 (37–48)		41 (38–47)		40 (36–46)		.195
LA surface, cm^2^, median (IQR)	22 (18–26)		22 (18–27)		21 (17–25)		21 (17–25)		.**025**
Moderate‐severe aortic stenosis, *n* (%)	62	(12.3)	52	(10.0)	51	(9.8)	35	(6.8)	.**030**
Moderate‐severe aortic regurgitation, *n* (%)	25	(5.0)	17	(3.3)	22	(4.2)	15	(2.9)	.312
Moderate‐severe mitral regurgitation, *n* (%)	77	(15.2)	65	(12.5)	65	(12.5)	43	(8.3)	.**008**
Moderate‐severe tricuspid regurgitation, *n* (%)	110	(21.8)	94	(18.1)	67	(12.9)	50	(9.7)	.**001**
Coronary angiography, *n* (%)	151	(29.9)	219	(42.3)	249	(47.8)	250	(48.4)	.**001**
No evidence of coronary artery disease	31	(20.5)	35	(16.0)	47	(18.9)	59	(23.6)	.329
1‐vessel disease	24	(15.9)	44	(20.1)	54	(21.7)	39	(15.6)	
2‐vessel disease	29	(19.2)	45	(20.5)	50	(20.1)	60	(24.0)	
3‐vessel disease	67	(44.4)	95	(43.4)	98	(39.4)	92	(36.8)	
Prior CABG	9	(6.0)	17	(7.8)	25	(10.0)	20	(8.0)	.532
Chronic total occlusion	21	(13.9)	35	(16.0)	29	(11.6)	23	(9.2)	.146
PCI, *n* (%)	83	(55.0)	127	(58.0)	138	(55.4)	118	(47.2)	.100
Sent to CABG, *n* (%)	8	(5.3)	16	(7.3)	14	(5.6)	11	(4.4)	.594
Baseline laboratory values, median (IQR)									
Potassium, mmol/L	3.9 (3.5–4.2)		3.9 (3.6–4.2)		3.9 (3.6–4.1)		3.9 (3.7–4.2)		.**005**
Sodium, mmol/L	139 (137–142)		139 (137–141)		139 (137–141)		139 (137–141)		.473
Albumin, g/L	24.4 (21.7–26.6)		30.6 (29.5–31.6)		34.1 (33.1–35.0)		38.0 (37.0–39.5)		.**001**
Urea, mg/dL	48.3 (31.8–72.1)		41.9 (30.5–61.4)		38.0 (29.2–51.6)		37.3 (29.2–48.2)		.**001**
Creatinine, mg/dL	1.20 (0.87–1.90)		1.08 (0.86–1.51)		1.04 (0.85–1.33)		1.04 (0.88–1.28)		.**001**
eGFR, mL/min/1.73 m^2^	56 (33–81)		61 (43–88)		69 (50–87)		70 (54–86)		.**001**
Hemoglobin, g/dL	10.1 (8.8–11.5)		12.0 (10.3–13.4)		13.0 (11.6–14.2)		14.0 (12.8–15.1)		.**001**
Iron, μg/dL	6.7 (5.0–10.4)		7.1 (5.0–11.0)		8.1 (6.1–13.7)		12.2 (8.0–16.7)		.**001**
Ferritin, ng/mL	271 (107–590)		96 (42–279)		116 (42–247)		95 (51–208)		.**001**
Transferrin, mg/dL	1.6 (1.3–2.1)		2.0 (1.7–2.6)		2.2 (1.9–2.5)		2.4 (2.1–3.0)		.**001**
Transferrin saturation, %	21 (15–31)		17 (13–26)		19 (13–26)		24 (18–30)		.**028**
WBC count, × 10^9^/L	8.90 (6.72–11.78)		8.25 (6.44–10.06)		8.00 (6.46–9.59)		7.95 (6.39–9.69)		.**001**
Platelet count, × 10^9^/L	246 (179–330)		227 (177–288)		219 (175–264)		222 (184–268)		.**001**
HbA1c, %	5.9 (5.4–7.0)		5.9 (5.5–7.0)		5.9 (5.5–6.6)		5.9 (5.5–6.7)		.973
Total cholesterol, mg/dL	133 (110–165)		150 (128–180)		162 (131–193)		173 (142–208)		.**001**
LDL‐cholesterol, mgl/dL	87 (66–110)		93 (71–120)		102 (75–125)		108 (81–140)		.**001**
HDL‐cholesterol, mg/dL	38 (28–48)		42 (34–53)		43 (35–51)		43 (36–53)		.**001**
C‐reactive protein, mg/L	47.2 (18.9–98.9)		18.2 (6.4–43.8)		8.8 (2.9–23.1)		3.2 (2.9–10.2)		.**001**
NT‐proBNP, pg/mL	5696 (2311–12857)		3037 (1390–6300)		1987 (980–4071)		1143 (297–2822)		.**001**
NT‐proBNP (eGFR corrected), pg/mL	2768 (1410–5818)		1713 (871–3548)		1538 (608–2831)		737 (283–1806)		.**001**
Cardiac troponin I, μg/L	0.07 (0.02–0.44)		0.05 (0.02–0.30)		0.02 (0.02–0.16)		0.02 (0.02–0.05)		.**001**
Medication at discharge, *n* (%)									
ACE‐inhibitor	211	(46.5)	251	(49.7)	284	(54.9)	269	(52.3)	.054
ARB	89	(19.6)	107	(21.2)	132	(25.5)	135	(26.3)	.**033**
Beta‐blocker	355	(78.2)	393	(77.8)	409	(79.1)	390	(75.9)	.648
Aldosterone antagonist	63	(13.9)	57	(11.3)	71	(13.7)	79	(15.4)	.294
ARNI	5	(1.1)	6	(1.2)	5	(1.0)	6	(1.2)	.987
SGLT2‐inhibitor	6	(1.3)	22	(4.4)	27	(5.2)	25	(4.9)	.**009**
Loop diuretics	299	(65.9)	249	(49.3)	226	(43.7)	180	(35.0)	.**001**
Statin	268	(59.0)	357	(70.7)	372	(72.0)	378	(73.5)	.**001**
Digitalis	25	(5.5)	28	(5.5)	27	(5.2)	18	(3.5)	.382
Amiodarone	14	(3.1)	12	(2.4)	18	(3.5)	11	(2.1)	.534
ASA	218	(48.0)	267	(52.9)	272	(52.6)	254	(49.4)	.341
P2Y12‐inhibitor	122	(26.9)	171	(33.9)	180	(34.8)	165	(32.1)	.**043**
DOAC	141	(31.1)	164	(32.5)	167	(32.3)	178	(34.6)	.688
Vitamin K antagonist	21	(4.6)	40	(7.9)	42	(8.1)	39	(7.6)	.127

*Note*: Level of significance *p* ≤ .05. Bold type indicates statistical significance.

Abbreviations: ACE, angiotensin‐converting enzyme; ADHF, acute decompensated heart failure; ARB, angiotensin receptor blocker; ARNI, angiotensin receptor neprilysin inhibitor; ASA, acetylsalicylic acid; CABG, coronary artery bypass grafting; DOAC, directly acting oral anticoagulant; eGFR, estimated glomerular filtration rate; HbA1c, glycated haemoglobin; HDL, high‐density lipoprotein; IQR, interquartile range; IVSd, interventricular septum in diastole; LA, left atrial; LDL, low‐density lipoprotein; LVEDD, Left ventricular end‐diastolic diameter; LVEF, left ventricular ejection fraction; NT‐proBNP, amino‐terminal prohormone of brain natriuretic peptide; NYHA, New York Heart Association; PCI, percutaneous coronary intervention; TAPSE, tricuspid annular plane systolic excursion; WBC, white blood cells.

In addition to the stratification by albumin levels, baseline characteristics and HF‐related as well as procedural data of the study cohort were also analysed based on the UAR and ACR quartiles. The corresponding results are presented in Tables S1 and S2 for the UAR and Tables S3 and S4 for the ACR.

### 3.2 Correlation of albumin, the UAR and ACR with clinical and laboratory data

As demonstrated in Table 3, albumin levels correlated with hemoglobin (*r* = .568), NT‐proBNP (*r* = −.448), and CRP levels (*r* = −.556). Furthermore, the UAR correlated with the eGFR (*r* = −.686), hemoglobin (*r* = −.441), and NT‐proBNP levels (*r* = .502) (*p* = .001 for all correlations). At last, the ACR was positively correlated to the eGFR (*r* = .869) and hemoglobin (*r* = .455), while being negatively correlated with NT‐proBNP (*r* = −.524).

**TABLE 3:** Spearman's rank correlation coefficients (*r*) of albumin, UAR, and ACR with laboratory as well as clinical parameters in all patients.

	Albumin (g/L)		UAR (mg/g)		ACR (g/mg)	
	*r*	*p*	*r*	*p*	*r*	*p*
Age (years)	−.187	.**001**	.337	.**001**	−.270	.**001**
Body mass index (kg/m^2^)	.168	.**001**	−.070	.**003**	.034	.**158**
LVEF (%)	.033	.**131**	−.059	.**007**	.043	.**051**
TAPSE (mm)	.067	.**002**	−.102	.**001**	.108	.**001**
eGFR (mL/min)	.168	.**001**	−.686	.**001**	.869	.**001**
Hemoglobin (g/dL)	.568	.**001**	−.441	.**001**	.455	.**001**
HbA1c (%)	.007	.**831**	.139	.**001**	−.174	.**001**
Platelet count (×10^9^/L)	−.098	.**001**	−.085	.**001**	.070	.**001**
NT‐proBNP (pg/mL)	−.448	.**001**	.502	.**001**	−.524	.**001**
Cardiac troponin I (μg/L)	−.272	.**001**	.161	.**001**	−.126	.**001**
C‐reactive protein (mg/L)	−.556	.**001**	.326	.**001**	−.335	.**001**

*Note*: Level of significance *p* < .05. Bold type indicates statistical significance and moderate to strong correlations considered in the case of *r* ≥ .4.

Abbreviations: ACR, albumin‐to‐creatinine ratio; eGFR, estimated glomerular filtration rate; HbA1c, glycated haemoglobin; LVEF, left ventricular ejection fraction; NT‐pro BNP, amino‐terminal prohormone of brain natriuretic peptide; TAPSE, tricuspid annular plane systolic excursion, UAR, urea‐to‐albumin ratio.

### 3.3 Prognostic impact of albumin levels, the UAR and the ACR in HFmrEF patients

**FIGURE 2:**
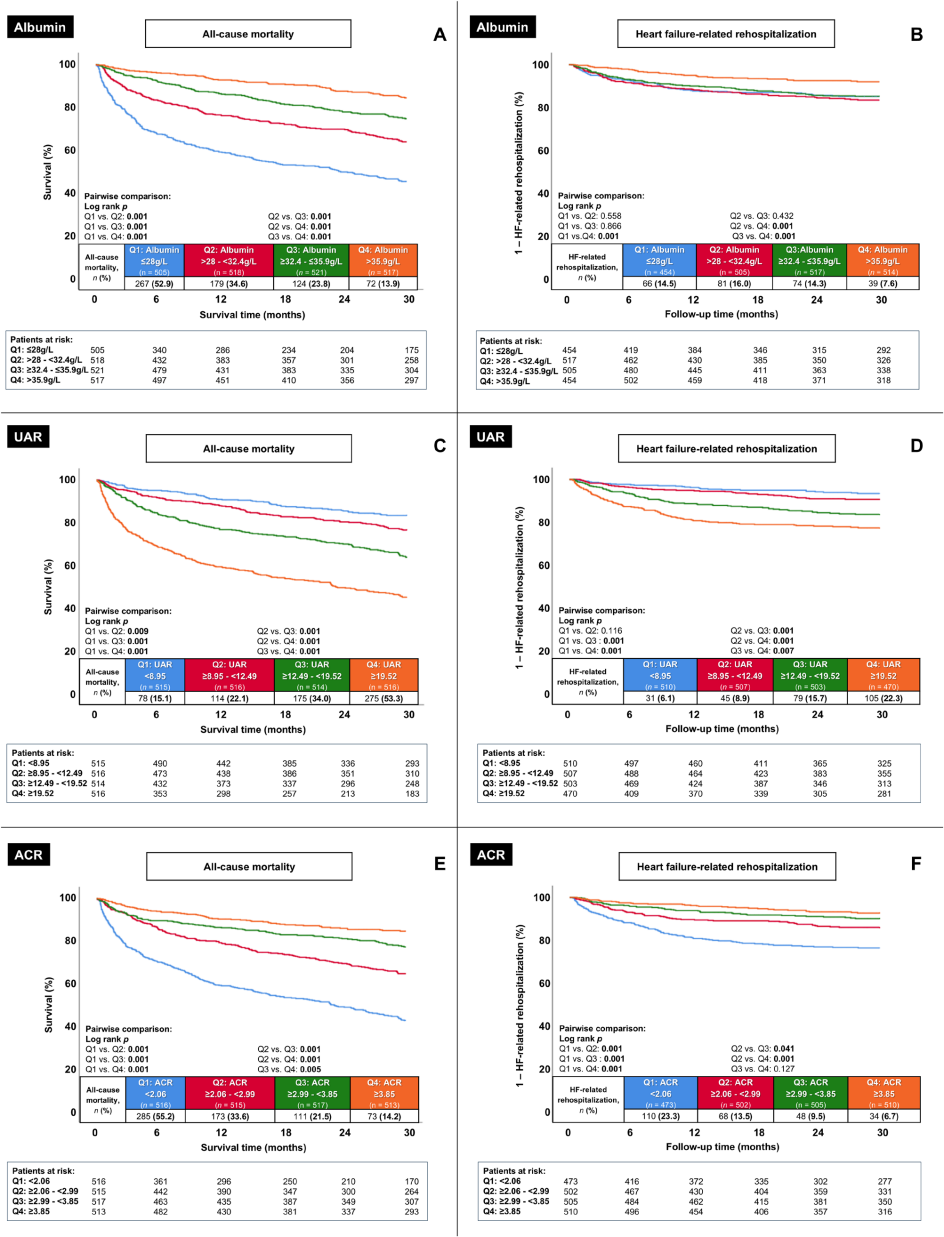
Kaplan Meier analyses displaying the prognostic impact of albumin levels (upper panels, A and B), the UAR (middle panels, C and D), and the ACR (lower panels, E and F) on long‐term all‐cause mortality (left panels) and HF‐related rehospitalization (right panels). ACR, albumin‐to‐creatinine ratio; HF, heart failure; UAR, urea‐to‐albumin ratio. Level of significance *p* ≤ 0.05. Bold type indicates statistical significance.

The median follow‐up time within the entire study cohort was 30 months (i.e. long‐term follow‐up). Analysis stratified by albumin quartiles demonstrated a continuous increase in the primary endpoint of long‐term all‐cause mortality with decreasing albumin levels (Q4: 13.9 vs. Q3: 23.8 vs. Q2: 34.6 vs. Q1: 52.9%; log rank *p* = .001 for all comparisons; Q3 vs. Q4: HR = 1.778; 95% CI 1.330–2.377; Q1 vs. Q4: HR = 5.269; 95% CI 4.061–6.838) (Figure 2, upper left panel; Table 4). Furthermore, patients in albumin Q4 showed a significantly lower rate of the secondary endpoint of HF‐related rehospitalization at 30 months, compared to Q1‐3 (Q4: 7.6 vs. Q3: 14.3 and Q2: 16.0 and Q1: 14.5%; log rank *p* = .001 for all comparisons with Q4; Q3 vs. Q4: HR = 1.943; 95% CI 1.318–2.863; Q1 vs. Q4: HR = 1.999; 95% CI 1.346–2.970) (Figure 2, upper right panel). Comparable results for the primary endpoint of long‐term all‐cause mortality were observed when the study cohort was stratified by UAR quartiles (Q1: 15.1 vs. Q2: 22.1 vs. Q3: 34.0 vs. Q4: 53.3%; log rank *p* ≤ .009 for all comparisons; Q2 vs. Q1: HR = 1.469; 95% CI 1.101–1.959; Q4 vs. Q1: HR = 4.718; 95% CI 3.668–6.068) (Figure 2, middle left panel) and ACR quartiles (Q1: 55.2 vs. Q2: 33.6 vs. Q3: 21.5 vs. Q4: 14.2%; log rank *p* ≤ .005 for all comparisons; Q3 vs. Q4: HR = 1.529; 95% CI 1.138–2.055; Q1 vs. Q4: HR = 5.164; 95% CI 3.992–6.680) (Figure 2, lower left panel).

**TABLE 4:** Follow‐up data, primary and secondary endpoints stratified by albumin levels, UAR and ACR.

	Albumin (g/L)								
	Q1: Albumin ≤28 g/L (*n* = 505)		Q2: Albumin >28–< 32.4 g/L (*n* = 518)		Q3: Albumin ≥32.4–≤ 35.9 g/L (*n* = 521)		Q4: Albumin >35.9 g/L (*n* = 517)		*p*
Primary endpoint, *n* (%)									
All‐cause mortality, at 30 months	267	(52.9)	179	(34.6)	124	(23.8)	72	(13.9)	.**001**
Secondary endpoints, *n* (%)									
All‐cause mortality, in‐hospital	51	(10.1)	13	(2.5)	4	(0.8)	3	(0.6)	.**001**
Cardiac mortality, in‐hospital	12	(2.4)	6	(1.2)	3	(0.6)	2	(0.4)	.**011**
Non‐cardiac mortality, in‐hospital	39	(7.7)	7	(1.4)	1	(0.2)	1	(0.2)	.**001**
All‐cause mortality, at 12 months	207	(41.0)	124	(23.9)	72	(13.8)	37	(7.2)	.**001**
Heart failure‐related rehospitalization, at 12 months	56	(12.3)	59	(11.7)	52	(10.1)	26	(5.1)	.**001**
Heart failure‐related rehospitalization, at 30 months	66	(14.5)	81	(16.0)	74	(14.3)	39	(7.6)	.**001**
Cardiac rehospitalization, at 30 months	89	(19.6)	124	(24.6)	126	(24.4)	99	(19.3)	.061
Coronary revascularization, at 30 months	12	(2.6)	36	(7.1)	41	(7.9)	47	(9.1)	.**001**
Acute myocardial infarction, at 30 months	14	(3.1)	15	(3.0)	21	(4.1)	10	(1.9)	.266
Stroke, at 30 months	11	(2.4)	18	(3.6)	10	(1.9)	16	(3.1)	.398
MACCE, at 12 months	225	(44.6)	159	(30.7)	104	(20.0)	80	(15.5)	.**001**
MACCE, at 30 months	283	(56.0)	219	(42.3)	167	(32.1)	126	(24.4)	.**001**
Follow‐up data, median (IQR)									
Hospitalization time, days	15 (10–27)		9 (6–16)		8 (5–12)		6 (4–9)		.**001**
ICU time, days	0 (0–2)		0 (0–1)		0 (0–1)		0 (0–0)		.**001**
Follow‐up time, days	481 (108–1207)		894 (318–1651)		1080 (510–1800)		1054 (617–1842)		.**001**

	UAR (mg/g)								
	Q1: UAR <8.95 mg/g (*n* = 515)		Q2: UAR ≥8.95–< 12.49 mg/g (*n* = 516)		Q3: UAR ≥12.49–< 19.52 mg/g (*n* = 514)		Q4: UAR ≥19.52 mg/g (*n* = 516)		*p* value
Primary endpoint, *n* (%)									
All‐cause mortality, at 30 months	78	(15.1)	114	(22.1)	175	(34.0)	275	(53.3)	.**001**
Secondary endpoints, *n* (%)									
All‐cause mortality, in‐hospital	5	(1.0)	9	(1.7)	11	(2.1)	46	(8.9)	.**001**
Cardiac mortality, in‐hospital	2	(0.4)	1	(0.2)	3	(0.6)	17	(3.3)	.**001**
Non‐cardiac mortality, in‐hospital	3	(0.6)	8	(1.6)	8	(1.6)	29	(5.6)	.**001**
All‐cause mortality, at 12 months	47	(9.1)	63	(12.2)	119	(23.2)	221	(40.9)	.**001**
Heart failure‐related rehospitalization, at 12 months	19	(3.7)	26	(5.1)	57	(11.3)	91	(19.4)	.**001**
Heart failure‐related rehospitalization, at 30 months	31	(6.1)	45	(8.9)	79	(15.7)	105	(22.3)	.**001**
Cardiac rehospitalization, at 30 months	81	(15.9)	115	(22.7)	121	(24.1)	121	(25.7)	.**001**
Coronary revascularization, at 30 months	35	(6.9)	47	(9.3)	37	(7.4)	17	(3.6)	.**006**
Acute myocardial infarction, at 30 months	14	(2.7)	15	(3.0)	13	(2.6)	18	(3.8)	.678
Stroke, at 30 months	13	(2.5)	19	(3.7)	13	(2.6)	10	(2.1)	.443
MACCE, at 12 months	82	(15.9)	107	(20.7)	151	(29.4)	228	(44.2)	.**001**
MACCE, at 30 months	122	(23.7)	167	(32.4)	213	(41.4)	293	(56.8)	.**001**
Follow‐up data, median (IQR)									
Hospitalization time, days	7 (4–11)		8 (5–13)		8 (6–14)		14 (8–23)		.**001**
ICU time, days	0 (0–1)		0 (0–1)		0 (0–1)		0 (0–1)		.270
Follow‐up time, days	1060 (536–1832)		1152 (537–1895)		870 (325–1553)		527 (103–1194)		.**001**

	ACR (g/mg)								
	Q1: ACR <2.06 g/mg (*n* = 516)		Q2: ACR ≥2.06–<2.99 g/mg (*n* = 515)		Q3: ACR ≥2.99– < 3.85 g/mg (*n* = 517)		Q4: ACR ≥3.85 g/mg (*n* = 513)		*p*
Primary endpoint, *n* (%)									
All‐cause mortality, at 30 months	285	(55.2)	173	(33.6)	111	(21.5)	73	(14.2)	.**001**
Secondary endpoints, *n* (%)									
All‐cause mortality, in‐hospital	43	(8.3)	13	(2.5)	12	(2.3)	3	(.6)	.**001**
Cardiac mortality, in‐hospital	14	(2.7)	6	(1.2)	3	(0.8)	0	(.0)	.**001**
Non‐cardiac mortality, in‐hospital	29	(5.6)	7	(1.4)	9	(1.7)	3	(.6)	.**001**
All‐cause mortality, at 12 months	212	(41.1)	108	(21.0)	71	(13.7)	49	(9.6)	.**001**
Heart failure‐related rehospitalization, at 12 months	91	(19.2)	52	(10.4)	31	(6.1)	19	(3.7)	.**001**
Heart failure‐related rehospitalization, at 30 months	110	(23.3)	68	(13.5)	48	(9.5)	34	(6.7)	.**001**
Cardiac rehospitalization, at 30 months	130	(27.5)	116	(23.1)	113	(22.4)	79	(15.5)	.**001**
Coronary revascularization, at 30 months	22	(4.7)	41	(8.2)	43	(8.5)	30	(5.9)	.**048**
Acute myocardial infarction, at 30 months	19	(4.0)	16	(3.2)	11	(2.2)	14	(2.7)	.390
Stroke, at 30 months	7	(1.5)	18	(3.6)	11	(2.2)	19	(3.7)	.087
MACCE, at 12 months	227	(44.0)	148	(28.7)	109	(21.1)	84	(16.4)	.**001**
MACCE, at 30 months	301	(58.3)	217	(42.1)	161	(31.1)	116	(22.6)	.**001**
Follow‐up data, median (IQR)									
Hospitalization time, days	14 (9–22)		9 (6–16)		7 (5–12)		7 (4–11)		.**001**
ICU time, days	0 (0–1)		0 (0–1)		0 (0–1)		0 (0–1)		.390
Follow‐up time, days	523 (118–1162)		928 (375–1627)		1104 (537–1897)		1054 (524–1814)		.**001**

*Note*: Level of significance *p* ≤ .05. Bold type indicates statistical significance.

Abbreviations: ACR, Albumin‐to‐creatinine ratio; ADHF, acute decompensated heart failure; CI, confidence interval; HF, heart failure; HR, hazard ratio; ICU, intensive care unit; IQR, interquartile range; MACCE, major adverse cardiac and cerebrovascular events; UAR, urea‐to‐albumin ratio.

However, a different pattern was observed regarding the secondary endpoint of HF‐related rehospitalization, whereas higher UAR quartiles were accompanied by a gradual increase in HF‐related rehospitalizations (Q1: 6.1 vs. Q2: 8.9 vs. Q3: 15.7 vs. Q4: 22.3%; Q2–4 vs. Q1: HR = 1.451, 2.686 and 4.018 with Q4 vs. Q1: 95% CI 2.691–5.998), which was statistically significant across all comparisons (log rank *p* = .001) except for the pairwise comparison between UAR Q1 and Q2 (log rank *p* = .116; HR = 1.451; 95% CI .918–2.292) (Figure 2, middle right panel). Similar results regarding the rate of long‐term HF‐related rehospitalization were observed for the ACR quartiles (Figure 2, lower right panel).

### 3.4 Multivariable Cox regression analyses

**FIGURE 3:**
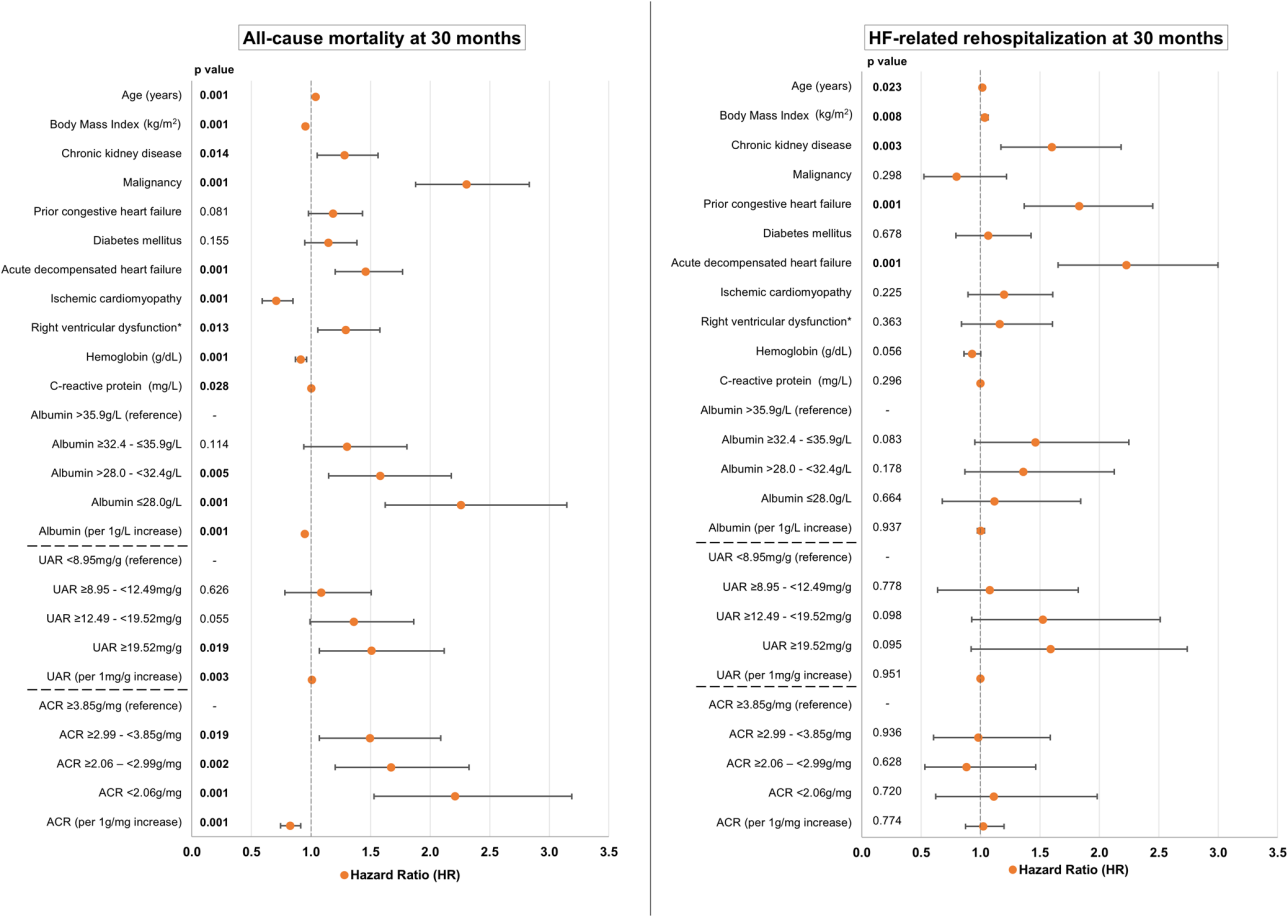
Forest plots demonstrating HRs and 95% CIs derived from multivariable Cox regression analyses regarding the risk of long‐term all‐cause mortality (left panel) and HF‐related rehospitalization (right panel) stratified by albumin levels, the UAR and the ACR during the index admission. ACR, albumin‐to‐creatinine ratio; CI, confidence interval; HF, heart failure; HR, hazard ratio; UAR, urea‐to‐albumin ratio. Level of significance *p* ≤ 0.05. Bold type indicates statistical significance. The multivariable model used to calculate the HRs and 95% CIs for the UAR and ACR quartile analyses, as well as the analysis using albumin, UAR and ACR as continuous variables, was adjusted for the same variables as in the categorical albumin quartile analysis. *Right ventricular dysfunction was defined as tricuspid annular plane systolic excursion <17 mm.

The association between lower albumin levels and a higher risk of long‐term all‐cause mortality was still observed after multivariable adjustment. In comparison to the albumin quartile Q4 (reference), the albumin quartiles Q1 and Q2 were associated with a significantly increased risk of the primary endpoint (Q1 vs. Q4: HR = 2.260; 95% CI 1.623–3.148 and Q2 vs. Q4: HR = 1.582; 95% CI 1.149–2.178), whereas Q3 vs. Q4 did not reach statistical significance (HR = 1.302; 95% CI .939–1.806; *p* = .114). Additionally, when included as a continuous variable (i.e. per 1 g/L increase), higher albumin levels were independently associated with a lower risk of long‐term all‐cause mortality (HR = .948; 95% CI .931–.966; *p* = .001). However, no association between albumin levels and the secondary endpoint of HF‐related rehospitalization was observed (*p* ≥ .083 within the categorical and continuous analysis) (Figure 3, albumin panel). Similar analyses were performed for the UAR and ACR. A higher UAR was also identified as an independent risk factor for long‐term all‐cause mortality after multivariable adjustment, although only achieving statistical significance when comparing UAR Q4 (reference) to Q1 (HR = 1.507; 95% CI 1.071–2.119; *p* = .019) and when investigated as a continuous variable (HR = 1.009; 95% CI 1.003–1.015; *p* = .003). The UAR was not significantly associated with the secondary endpoint of long‐term HF‐related rehospitalization after multivariable adjustment (*p* ≥ .095 for the categorical and continuous analysis of the UAR) (Figure 3, UAR panels). The ACR was the only parameter of the three that demonstrated a significant association with all‐cause mortality for all quartile comparisons (i.e. Q1, Q2 and Q3 vs. Q4) following multivariable adjustment (Q1 vs. Q4: HR = 2.208; 95% CI 1.528–3.190 and Q2 vs. Q4: HR = 1.672; 95% CI 1.202–2.326). Nonetheless, there was no association between the ACR and long‐term HF‐related rehospitalization after multivariable adjustment (*p* ≥ .628 for all quartile comparisons).

Post‐hoc power analyses based on Schoenfeld's method were performed for the primary endpoint. The detectable HRs for the Q1 vs. Q4 contrast were 1.80 (80% power) and 1.97 (90% power) for albumin, 1.79 and 1.95 for UAR, and 1.34 and 1.40 for ACR. The observed adjusted HRs (Q1 vs. Q4) were 2.26 for albumin, 1.51 for UAR, and 2.21 for ACR. Thus, the analyses for albumin and ACR were adequately powered to detect the observed effects, whereas the UAR analysis was potentially underpowered given that its observed HR was smaller than the minimum detectable thresholds.

Multivariable Cox regression analyses regarding the primary endpoint were additionally performed within specific subgroups stratified by eGFR (i.e. >60, ≤60–>30, and ≤30 mL/min), the presence of diabetes mellitus and prior congestive HF (Figure S1). Lower albumin levels were associated with an increased risk of all‐cause mortality, particularly in patients with a moderate decrease of their eGFR (i.e. ≤60 mL/min).

## 4 DISCUSSION

This study investigated the prognostic impact of albumin, the UAR, and the ACR in consecutive patients hospitalized with HFmrEF using a large retrospective dataset. Stratification into quartiles revealed that patients with lower albumin, higher UARs, and lower ACRs were older, had more comorbidities, poorer nutritional status, and a higher prevalence of concomitant anemia. Furthermore, lower albumin levels, higher UARs and lower ACRs were associated with a linear increase in the risk of long‐term all‐cause mortality. The prognostic impact of the three investigated parameters on the primary endpoint of long‐term all‐cause mortality was confirmed using multivariable Cox regression analyses, with the ACR showing the most robust association with increased mortality across all quartile comparisons. However, neither albumin, the UAR, nor the ACR were associated with the key secondary endpoint of long‐term HF‐related rehospitalization after multivariable adjustment, which may partly relate to limited event numbers. HFmrEF was intentionally selected as the target population because it is increasingly acknowledged as its own HF phenotype in contemporary guidelines but remains insufficiently characterized. Its overlap with HFpEF and HFrEF creates clinical uncertainty, making focused evaluation of prognostic factors particularly valuable. Investigating this group directly supports better risk stratification and management in a population where evidence remains sparse.

Albumin has become an established prognostic marker in cardiovascular diseases (e.g. myocardial infarction,^24^ stroke^25^ and chronic as well as acute HF^14^, ^26^), while its predictive value in the specific subgroup of HFmrEF remains inconclusive. A recent meta‐analysis by El Iskandarani et al.^13^ estimated that hypoalbuminemia affects 12%–54% of HF patients, with higher rates in those with acute HF, likely due to fluid retention and volume overload.^13^ This aligns with the present study, where ADHF was more common in patients with lower albumin levels. Notably, almost 70% of the cohort had hypoalbuminemia, which may be explained by the study setting (i.e. exclusively hospitalized patients who typically present with greater systemic inflammation, fluid overload, and nutritional deficits), the less stringent definition of hypoalbuminemia (i.e. albumin <35 g/L), and the cohorts advanced age together with a high burden of comorbidity, particularly CKD. Most recently, Biancucci et al.^27^ provided a comprehensive review confirming the consistent association between hypoalbuminemia and adverse outcomes in HF. Summarizing the two existing meta‐analyses on the topic,^13^, ^15^ they reported that low serum albumin was associated with a significantly higher risk of both short‐ and long‐term mortality across HF populations, even after adjusting for confounders such as sex, BMI, CKD and CRP levels. These results are consistent with the present study and are further supported by evidence from the TOPCAT^28^ and EMPEROR‐Preserved^29^ trials, which additionally observed that low albumin levels were associated with greater arterial stiffness, diastolic dysfunction, pulmonary hypertension, and biomarkers indicative of inflammation and liver fibrosis.^28^, ^29^ Moreover, the recently published ALBIMED‐HF study^30^ further confirmed the strong prognostic value of hypoalbuminemia in patients hospitalized exclusively for acute HF, demonstrating an independent and highly significant association with increased 12‐month all‐cause mortality. These findings reinforce the prognostic relevance of hypoalbuminemia across different HF phenotypes and clinical settings.

In additive alignment with the present study, the available evidence does not indicate a strong link between albumin levels and hospital readmission rates, despite albumins critical role in maintaining fluid balance.^13^ The reasons for this lack of association remain unclear, though several factors may contribute. Low albumin levels often indicate systemic inflammation and general morbidity, which are more closely tied to mortality than HF‐specific hospitalization. Additionally, patients with low albumin may receive closer medical supervision due to perceived frailty, resulting in better outpatient management and potentially reducing HF‐related rehospitalizations. Notably, only 4% of patients in the meta‐analysis by El Iskandarani et al.^13^ were derived from European studies, just 21% had chronic rather than acute HF, and LVEF‐based stratification was rarely conducted. This underscores the prognostic value of the present study, which focused on patients hospitalized with both chronic and acute HFmrEF.

Furthermore, the prognostic benefit of interventions (e.g. albumin substitution or structured nutrition programs) addressing low albumin levels in HF patients remains unclear. On the one hand, clinical studies have reported that an increase in albumin levels during hospitalization for ADHF was associated with a reduced risk of adverse events post‐admission.^31^, ^32^ On the other hand, intravenous substitution of albumin was not observed to improve the prognosis of patients with acute HF and was associated with higher in‐hospital mortality in ICU patients with congestive HF, even after adjusting for confounders.^33^, ^34^ This may be explained by previous studies indicating that oedema formation, driven by increased vascular endothelial permeability, is primarily influenced by hydrostatic venous pressure rather than colloid oncotic pressure, limiting the effects of albumin substitution.^35^ Additionally, albumin infusion may increase cardiac workload, further complicating outcomes. The EFFORT trial demonstrated that protocol‐guided nutritional support reduced mortality and major cardiovascular events in hospitalized patients with chronic HF.^36^ However, a secondary analysis of EFFORT revealed that nutritional support did not significantly raise short‐term albumin levels, and changes in albumin were not indicative of a response to nutritional interventions.^37^

In addition, the authors aimed to explore albumin‐derived ratios potentially offering greater prognostic value than albumin alone. While elevated urea reflects reduced renal perfusion or increased protein catabolism, albumin is more indicative of chronic inflammation and impaired protein synthesis. Thus, the authors hypothesized that the UAR could integrate systemic inflammation, protein metabolism, and renal dysfunction into a single metric, providing a more comprehensive representation of the physiological state of HF patients. This hypothesis aligns with evidence from Novack et al.,^38^ which identified hypoalbuminemia and elevated urea levels as top predictors of one‐year mortality in patients with ADHF. Similarly, combining albumin with creatinine may provide enhanced prognostic value, as creatinine serves as a marker of muscle mass and reflects renal function more reliably than urea.^39^

To date, three studies^40^, ^41^, ^42^ have investigated the prognostic value of the BUN‐to‐albumin ratio in HF patients, whereas none have examined the UAR, as BUN resembled the standard laboratory measure in their clinical setting. Since BUN and urea reflect similar (patho‐)physiological processes, evidence on the BUN‐to‐albumin ratio should be discussed. This ratio was consistently identified as a strong predictor of mortality in HF, with HRs of two to three in patients with elevated values.^40^, ^41^, ^42^ These findings align with the present study, where higher UARs also predicted increased mortality, even after adjusting for confounders such as CKD, malignancy, hemoglobin or CRP levels. However, prior studies were limited by their specific cohort^40^ (i.e. critically ill patients treated on the ICU), exclusion criteria^40^, ^42^ (e.g. malignancies and renal dysfunction), or short follow‐up (i.e. 90 days).^40^, ^41^ Notably, only one study investigated rehospitalization, with supplementary analyses suggesting a weak association with the BUN‐to‐albumin ratio.^41^ The present study investigated long‐term HF‐related rehospitalization as a key secondary endpoint but also found no significant association with the UAR, suggesting its predictive value may be influenced by factors like age and CKD, which were adjusted for within multivariable regression analyses.

Similar to the UAR, the blood‐derived ACR has not been extensively studied. Data from Li et al.^43^ indicate ACRs predictive value for mortality in a general HF cohort, with additional studies linking low ACR to adverse outcomes in specific patient populations (i.e. HF patients treated in the ICU,^44^ suffering from comorbid CKD,^45^ or heart transplant recipients^46^). Furthermore, Guo et al.^47^ conducted the only study to date investigating the ACRs predictive value for HF‐related rehospitalization. In contrast to the present study, they observed that higher ACRs reduced the risk of readmission at 28 days and 3 as well as 9 months. However, their cohort exclusively included elderly HF patients (≥60 years) without stratification by LVEF, which may explain the differing results. Lastly, prior studies did not assess the prognostic value of albumin levels in comparison to the investigated albumin‐derived ratio. Consequently, it remains uncertain whether the ratios offered a predictive advantage over the isolated consideration of albumin within their cohorts. The limited incremental prognostic value of UAR and ACR beyond albumin alone within the present study may be explained by the weaker association of urea and creatinine with long‐term outcomes in HFmrEF, where renal dysfunction is less pronounced than in HFrEF.^48^, ^49^ Furthermore, albumin already integrates multiple systemic processes relevant to HF pathophysiology, including inflammation, hepatic and renal congestion, and nutritional status, thereby potentially encompassing much of the prognostic information conveyed by these ratios.

### 4.1 Strengths and limitations of this study

A key strength of the present study is its explicit focus on HFmrEF, including only patients with standardized echocardiographic confirmation of the diagnosis, addressing a previously understudied cohort. In addition, the study's comprehensive data collection from multiple sources (e.g. charts, lab findings, technical examinations) offers greater accuracy than studies relying on International Statistical Classification of Diseases and Related Health Problems (ICD) codes. Consequently, the HARMER registry data may better reflect the true prevalence of cardiac and non‐cardiac comorbidities, which are crucial for interpreting albumin, urea, and creatinine levels. Lastly, the minimal lost‐to‐follow‐up rate with regard to the primary endpoint ensured robust data on long‐term mortality.

However, certain limitations must be acknowledged. The retrospective, single‐centre design may introduce confounding factors and limit generalizability. In addition, it restricted the study's sample size and event numbers, which might have resulted in insufficient statistical power to detect weaker prognostic associations in secondary endpoints. Pharmacological treatment after the index admission was not standardized, potentially influencing outcomes. Detailed nutritional and functional parameters beyond BMI, iron status, cholesterol levels, and NYHA classification were not available, limiting the ability to comprehensively characterize the nutritional status of patients included in the present cohort. Moreover, assessing the prognostic impact of nutritional interventions or albumin substitution was beyond the scope of this study. Additionally, causes of death beyond those occurring in the index hospitalization could not be obtained, preventing analysis of non‐cardiovascular mortality. Patient stratification was based on the measurement of albumin, urea, and creatinine collected closest to the index echocardiography. This time point was chosen as it represents a clinically stabilized phase where patients were sufficiently recompensated to undergo structural cardiac assessment, thereby ensuring temporal alignment between biochemical and cardiac data. However, the retrospective design of the study made it unfeasible to standardize measurement time points to account for fluctuations occurring during admission or follow‐up.

## 5 CONCLUSIONS

Hypoalbuminemia is common in HFmrEF, affecting two out of three patients in the present cohort. Low albumin levels, ACRs, and elevated UARs consistently predicted the risk of long‐term all‐cause mortality even after adjusting for potential confounders. None of the three markers predicted HF‐related rehospitalization after multivariable adjustment. The UAR and ACR did not provide a clinically significant predictive advantage over albumin levels alone.

## AUTHOR CONTRIBUTIONS

Conceptualization: A.S., I.A., M.A., K.W., T.B., D.D., M.B., T.S. Data curation: A.S., M.R., N.A., F.L., J.D., M.A., T.S. Formal analysis: A.S., I.A., M.A., K.W., T.B., D.D., M.B., T.S. Investigation: A.S., I.A., M.R., N.A., F.L., J.D., M.A., K.W., M.B., T.S. Methodology: A.S., I.A., M.A., K.W., T.B., D.D., M.B., T.S. Project administration: I.A., T.B., D.D., M.B., T.S. Resources: I.A., D.D., M.B., T.S. Software: I.A., D.D., M.B., T.S. Supervision: I.A., T.B., D.D., M.B., T.S. Validation: A.S., I.A., T.B., D.D., M.B., T.S. Visualization: A.S., M.R., N.A., F.L., J.D., T.S. Writing – original draft: A.S., I.A., M.B., T.S. Writing – review and editing: A.S., I.A., M.R., N.A., F.L., J.D., M.A., K.W., T.B., D.D., M.B., T.S.

## FUNDING INFORMATION

No funding was received for the research presented in this manuscript.

## CONFLICT OF INTEREST STATEMENT

The authors declare no conflicts of interest.

## CONSENT FOR PUBLICATION

The authors confirm that patient consent is not applicable to this article because solely retrospective data assembled in the daily clinical routine were used. In addition, pseudonymization of all patient‐related data was conducted prior to statistical analysis.

## Supporting information


**Data S1.** Supporting Information.
**Figure S1.** Forest plots demonstrating HRs and 95% CIs derived from multivariable Cox regression analyses regarding the risk of long‐term all‐cause mortality stratified by albumin levels (left panel, A), the UAR (middle panel, B), and ACR (right panel, C) within specific subgroups defined by eGFR and the presence or absence of diabetes mellitus or a prior diagnosis of congestive HF. ACR, albumin‐creatinine ratio; CI, confidence interval; HF, heart failure; HR, hazard ratio; UAR, urea‐to‐albumin ratio. Level of significance *p* ≤ .05. Bold type indicates statistical significance. The multivariable model used to calculate the HRs and 95% CIs for the albumin, UAR, and ACR quartile analyses was adjusted for the same variables as listed in the main multivariable Cox regression model displayed in Figure 3.

## Data Availability

The datasets used and/or analyzed for the current study are available from the corresponding author on reasonable request.
