# Supplementary material for: Albumin, urea‐to‐albumin ratio, or the albumin‐to‐creatinine ratio to predict outcomes in heart failure with mildly reduced ejection fraction
Source: Eur J Clin Invest. 2025 Dec 26;56(1):e70165. doi: 10.1111/eci.70165 (PMC12827846; doi:10.1111/eci.70165)
Supplement: Supplementary file 1 — Data S1. Supporting Information. Figure S1. Forest plots demonstrating HRs and 95% CIs derived from multivariable Cox regression analyses regarding the risk of long‐term all‐cause mortality stratified by albumin levels (left panel, A), the UAR (middle panel, B), and ACR (right panel, C) within specific subgroups defined by eGFR and the presence or absence of diabetes mellitus or a prior diagnosis of congestive HF. ACR, albumin‐creatinine ratio; CI, confidence interval; HF, heart failure; HR, hazard ratio; UAR, urea‐to‐albumin ratio. Level of significance p ≤ .05. Bold type indicates statistical significance. The multivariable model used to calculate the HRs and 95% CIs for the albumin, UAR, and ACR quartile analyses was adjusted for the same variables as listed in the main multivariable Cox regression model displayed in Figure 3. [file ECI-56-e70165-s001.zip › eci70165-sup-0001-AppendixS1.pdf]

Albumin, Urea-to-Albumin Ratio or the Albumin-to-Creatinine Ratio to Predict Outcomes in Heart Failure with Mildly Reduced Ejection Fraction

Alexander Schmitt* MS[1]; Ibrahim Akin* MD[1]; Marielen Reinhardt MS[1]; Noah Abel MS[1]; Felix Lau MS[1]; Jonas Dudda MD[1]; Mohammad Abumayyaleh MD[1]; Kathrin Weidner MD[1]; Thomas Bertsch MD[2]; Daniel Duerschmied MD[1]; Michael Behnes MD[1] and Tobias Schupp MD[1]

[1] Department of Cardiology, Angiology, Haemostaseology and Medical Intensive Care, University Medical Centre Mannheim, Medical Faculty Mannheim, Heidelberg University, Germany.

[2] Institute of Clinical Chemistry, Laboratory Medicine and Transfusion Medicine, Nuremberg General Hospital, Paracelsus Medical University, Nuremberg, Germany.

*Alexander Schmitt and Ibrahim Akin contributed equally.

Supplemental Material

(intended for publication as an online data supplement)

Supplemental Tables 1-5 and Supplemental Figure 1

Supplemental Table 1 - Baseline characteristics.

	**Q1: UAR <8.95mg/g** (*n* = 515)		**Q2: UAR ≥8.95 - <12.49mg/g** (*n* = 516)		**Q3: UAR ≥12.49 - <19.52mg/g** (*n* = 514)		**Q4: UAR ≥19.52mg/g** (*n* = 516)		**p value**
**Age**, median (IQR)	67	(54-77)	74	(63-82)	78	(69-84)	80	(72-85)	**0.001**
**Male sex**, n (%)	327	(63.5)	348	(67.4)	324	(63.0)	332	(64.3)	0.447
**Body mass index,** kg/m^2, median (IQR)	27.4	(24.1-31.2)	26.4	(24.2-30.1)	26.6	(23.8-30.9)	26.0	(23.0-30.4)	**0.009**
**SBP**, mmHg, median (IQR)	146	(130-166)	145	(130-167)	140	(121-160)	139	(118-160)	**0.001**
**DBP**, mmHg, median (IQR)	83	(72-95)	80	(71-92)	77	(69-88)	73	(63-86)	**0.001**
**Heart rate**, bpm, median (IQR)	82	(69-95)	80	(68-93)	80	(68-96)	81	(69-96)	0.533
**Medical history**, n (%)									
Coronary artery disease	165	(32.0)	202	(39.1)	227	(44.2)	245	(47.5)	**0.001**
Prior myocardial infarction	96	(18.6)	139	(26.9)	110	(21.4)	140	(27.1)	**0.002**
Prior PCI	121	(23.5)	143	(27.7)	147	(28.6)	159	(30.8)	0.064
Prior CABG	24	(4.7)	48	(9.3)	63	(12.3)	68	(13.2)	**0.001**
Prior valvular surgery	19	(3.7)	22	(4.3)	28	(5.4)	22	(4.3)	0.573
Congestive heart failure	117	(22.7)	147	(28.5)	184	(35.8)	240	(46.5)	**0.001**
Prior LVEF available, n (%)	156	(30.3)	163	(31.6)	184	(35.8)	234	(45.3)	**0.001**
Prior LVEF, median (IQR)	51	(44-60)	50	(45-60)	50	(41-60)	51	(43-60)	0.772
Prior LVEF ≥50%	87	(55.8)	93	(57.1)	96	(52.2)	135	(57.7)	0.700
Prior LVEF 41-49%	39	(25.0)	34	(20.9)	42	(22.8)	45	(19.2)	0.563
Prior LVEF ≤40%	12	(7.7)	15	(9.2)	23	(12.5)	25	(10.7)	0.498
Decompensated heart failure within 12 months prior to index	27	(5.2)	37	(7.2)	61	(11.9)	92	(17.8)	**0.001**
Prior ICD	9	(1.7)	8	(1.6)	15	(2.9)	8	(1.6)	0.320
Prior sICD	4	(0.8)	0	(0.0)	0	(0.0)	4	(0.8)	**0.046**
Prior CRT-D	4	(0.8)	5	(1.0)	9	(1.8)	11	(2.1)	0.205
Prior Pacemaker	34	(6.6)	37	(7.2)	54	(10.5)	63	(12.2)	**0.004**
Chronic kidney disease	23	(4.5)	64	(12.4)	201	(39.1)	360	(69.8)	**0.001**
Peripheral artery disease	29	(5.6)	58	(11.2)	55	(10.7)	88	(17.1)	**0.001**
Stroke	72	(14.0)	78	(15.1)	89	(17.3)	79	(15.3)	0.518
Liver cirrhosis	3	(0.6)	5	(1.0)	19	(3.7)	16	(3.1)	**0.001**
Malignancy	49	(9.5)	66	(12.8)	78	(15.2)	118	(22.9)	**0.001**
COPD	40	(7.8)	48	(9.3)	66	(12.8)	93	(18.0)	**0.001**
**Cardiovascular risk factors,** n (%)									
Arterial hypertension	364	(70.7)	397	(76.9)	411	(80.0)	436	(84.5)	**0.001**
Diabetes mellitus	139	(27.0)	174	(33.7)	197	(38.3)	248	(48.1)	**0.001**
Hyperlipidemia	148	(28.7)	163	(31.6)	152	(29.6)	164	(31.8)	0.648
Smoking	237	(46.0)	201	(39.0)	155	(30.2)	165	(32.0)	**0.001**
Current	153	(29.7)	108	(20.9)	68	(13.2)	62	(12.0)	**0.001**
Former	84	(16.3)	93	(18.0)	87	(16.9)	103	(20.0)	0.438
Family history	72	(14.0)	55	(10.7)	37	(7.2)	25	(4.8)	**0.001**
**Comorbidities at index hospitalization**, n (%)									
Acute coronary syndrome									
Unstable angina	30	(5.8)	44	(8.5)	13	(2.5)	9	(1.7)	**0.001**
STEMI	53	(10.3)	46	(8.9)	52	(10.1)	18	(3.5)	**0.001**
NSTEMI	63	(12.2)	69	(13.4)	63	(12.3)	72	(14.0)	0.802
Acute decompensated heart failure	42	(8.2)	72	(14.0)	123	(23.9)	229	(44.4)	**0.001**
Cardiogenic shock	9	(1.7)	12	(2.3)	10	(1.9)	21	(4.1)	0.070
Atrial fibrillation	166	(32.2)	198	(38.4)	233	(45.3)	262	(50.8)	**0.001**
Cardiopulmonary resuscitation	8	(1.6)	18	(3.5)	10	(1.9)	15	(2.9)	0.171
Out-of-hospital	4	(0.8)	9	(1.7)	4	(0.8)	5	(1.0)	0.376
In-hospital	4	(0.8)	9	(1.7)	6	(1.2)	10	(1.9)	0.368
Stroke	93	(18.1)	89	(17.2)	59	(11.5)	57	(11.0)	**0.001**
**Medication on admission**, n (%)									
ACE-inhibitor	151	(29.3)	196	(38.0)	215	(41.8)	176	(34.1)	**0.001**
ARB	90	(17.5)	111	(21.5)	119	(23.2)	135	(26.2)	**0.008**
Beta-blocker	243	(47.2)	258	(50.0)	308	(59.9)	354	(68.6)	**0.001**
Aldosterone antagonist	28	(5.4)	32	(6.2)	64	(12.5)	61	(11.8)	**0.001**
ARNI	2	(0.4)	3	(0.6)	4	(0.8)	7	(1.4)	0.319
SGLT2-inhibitor	10	(1.9)	14	(2.7)	12	(2.3)	6	(1.2)	0.333
Loop diuretics	97	(18.8)	127	(24.6)	223	(43.4)	322	(62.4)	**0.001**
Statin	194	(37.7)	235	(45.5)	248	(48.2)	254	(49.2)	**0.001**
ASA	155	(30.1)	185	(35.9)	178	(34.6)	176	(34.1)	0.231
P2Y12-inhibitor	36	(7.0)	56	(10.9)	58	(11.3)	44	(8.5)	0.061
DOAC	103	(20.0)	113	(21.9)	132	(25.7)	136	(26.4)	**0.047**
Vitamin K antagonist	32	(6.2)	35	(6.8)	48	(9.3)	62	(12.0)	**0.003**

ACE, angiotensin-converting-enzyme; ARB, angiotensin receptor blocker; ARNI, angiotensin receptor neprilysin inhibitor; ASA, acetylsalicylic acid; CABG, coronary artery bypass grafting; CKD, chronic kidney disease; COPD, chronic obstructive pulmonary disease; CRT-D, cardiac resynchronization therapy with defibrillator; DBP, diastolic blood pressure; DOAC, directly acting oral anticoagulant; IQR, interquartile range; PCI, percutaneous coronary intervention; (N)STEMI, non-ST-segment elevation myocardial infarction; SBP, systolic blood pressure; SGLT2, sodium glucose linked transporter 2; (s) ICD, (subcutaneous) implantable cardioverter defibrillator; UAR, urea-to-albumin ratio. Level of significance p≤0.05. Bold type indicates statistical significance.

Supplemental Table 2 - Heart failure-related and procedural data.

	Q1: UAR <8.95mg/g (*n* = 515)	Q2: UAR ≥8.95 - <12.49mg/g (*n* = 516)	Q3: UAR ≥12.49 - <19.52mg/g (*n* = 514)	Q4: UAR ≥19.52mg/g (*n* = 516)	p value
**Heart failure etiology**, n (%)					
Ischemic cardiomyopathy	278 (54.0)	303 (58.7)	312 (60.7)	298 (57.8)	
Non-ischemic cardiomyopathy	41 (8.0)	33 (6.4)	36 (7.0)	37 (7.2)	
Hypertensive cardiomyopathy	51 (9.9)	42 (8.1)	35 (6.8)	36 (7.0)	
Congenital heart disease	2 (0.4)	1 (0.2)	0 (0.0)	1 (0.2)	
Valvular heart disease	17 (3.3)	18 (3.5)	27 (5.3)	31 (6.0)	0.191
Tachycardia associated	28 (5.4)	36 (7.0)	24 (4.7)	32 (6.2)	
Tachymyopathy	11 (2.1)	8 (1.6)	8 (1.6)	9 (1.7)	
Pacemaker-induced cardiomyopathy	3 (0.6)	5 (1.0)	2 (0.4)	9 (1.7)	
Unknown	95 (18.4)	78 (15.1)	78 (15.2)	72 (14.0)	
**NYHA functional class,** n (%)					
I/II	440 (85.4)	415 (80.4)	353 (68.7)	291 (56.4)	
III	57 (11.1)	79 (15.3)	114 (22.2)	135 (26.2)	**0.001**
IV	18 (3.5)	22 (4.3)	47 (9.1)	90 (17.4)	
**Echocardiographic data**					
LVEF, %, median (IQR)	45 (45-47)	45 (45-47)	45 (45-47)	45 (45-47)	**0.034**
IVSd, median (IQR)	11 (10-13)	12 (11-13)	12 (11- 13)	12 (11-13)	0.077
LVEDD, mm, median (IQR)	49 (44-54)	49 (44-53)	48 (44-54)	50 (45-55)	0.203
TAPSE, mm, median (IQR)	20 (18-23)	20 (18-23)	20 (17-22)	19 (16-23)	**0.001**
LA diameter, mm, median (IQR)	40 (35-45)	41 (36-46)	42 (38-48)	44 (39-49)	**0.001**
LA surface, cm^2, median (IQR)	19 (16-22)	21 (17-25)	22 (18-25)	24 (20-29)	**0.001**
Moderate-severe aortic stenosis, n (%)	24 (4.7)	48 (9.3)	60 (11.7)	68 (13.2)	**0.001**
Moderate-severe aortic regurgitation, n (%)	12 (2.3)	14 (2.7)	22 (4.3)	31 (6.0)	**0.008**
Moderate-severe mitral regurgitation, n (%)	38 (7.4)	50 (9.7)	71 (13.8)	91 (17.6)	**0.001**
Moderate-severe tricuspid regurgitation, n (%)	41 (8.0)	60 (11.6)	89 (17.3)	131 (25.4)	**0.001**
**Coronary angiography,** n (%)	255 (49.5)	253 (49.0)	210 (40.9)	151 (29.3)	**0.001**
No evidence of coronary artery disease	65 (25.5)	49 (19.4)	29 (13.8)	29 (19.2)	
1-vessel disease	50 (19.6)	46 (18.2)	47 (22.4)	18 (11.9)	**0.003**
2-vessel disease	56 (22.0)	52 (20.6)	51 (24.3)	25 (16.6)	
3-vessel disease	84 (32.9)	106 (41.9)	83 (39.5)	79 (52.3)	
Prior CABG	14 (5.5)	17 (6.7)	24 (11.4)	16 (10.6)	0.063
Chronic total occlusion	25 (9.8)	31 (12.3)	27 (12.9)	25 (16.6)	0.259
PCI, n (%)	136 (53.3)	125 (49.4)	131 (62.4)	74 (49.0)	**0.022**
Sent to CABG, n (%)	11 (4.3)	21 (8.3)	8 (3.8)	9 (6.0)	0.135
**Baseline laboratory values**, median (IQR)					
Potassium, mmol/L	3.8 (3.6-4.0)	3.9 (3.6-4.1)	3.9 (3.6 (4.2)	4.0 (3.6-4.3)	**0.001**
Sodium, mmol/L	139 (137-141)	139 (137-141)	139 (137-141)	139 (137-142)	0.554
Albumin, g/dL	3.50 (3.21-3.78)	3.41 (3.07-3.69)	3.16 (2.80-3.48)	2.80 (2.38-3.20)	**0.001**
Urea, mg/dL	24.7 (20.6-28.5)	35.6 (31.8-39.3)	47.7 (42.2-54.2)	82.4 (63.8-115.4)	**0.001**
Creatinine, mg/dL	0.87 (0.73-1.00)	0.99 (0.85-1.17)	1.16 (0.95-1.46)	1.84 (1.32-2.80)	**0.001**
eGFR, mL/min/1.73 m^2	87 (72-102)	74 (60-88)	58(45-73)	33 (21-51)	**0.001**
Hemoglobin, g/dL	13.6 (12.0-14.7)	13.2 (11.5-14.5)	12.0 (10.5-13.6)	10.4 (9.1-12.2)	**0.001**
Iron, µg/dL	7.6 (5.4-15.6)	8.6 (5.0-14.1)	7.7 (5.0-11.9)	7.3 (5.1-11.1)	0.642
Ferritin, ng/mL	141 (58-325)	97 (47-208)	121 (44-344)	188 (67-440)	**0.021**
Transferrin, mg/dL	2.1 (1.7-2.5)	2.0 (1.7-2.6)	2.2 (1.6-2.6)	1.9 (1.5-2.3)	0.080
Transferrin saturation, %	20 (13-25)	23 (18-31)	18 (13-26)	19 (14-29)	0.328
WBC count, x 10^9/L	8.09 (6.46-9.65)	8.05 (6.39-9.64)	7.98 (6.28-9.99)	8.95 (6.88-11.64)	**0.001**
Platelet count, x 10^9/L	232 (194-282)	234 (188-291)	218 (169-271)	222 (167-291)	**0.001**
HbA1c, %	5.7 (5.4-6.4)	5.9 (5.5-6.7)	5.9 (5.5-6.8)	6.1 (5.6-7.4)	**0.001**
Total cholesterol, mg/dL	171 (139-198)	164 (132-197)	148 (122-177)	138 (113-165)	**0.001**
LDL-cholesterol, mg/dL	110 (82-139)	101 (78-130)	93 (71-115)	81 (61-110)	**0.001**
HDL-cholesterol, mg/dL	42 (35-53)	43 (36-52)	42 (34-52)	40 (32-48)	**0.008**
C-reactive protein, mg/L	7 (3-21)	7 (3-25)	16 (5-48)	33 (11-81)	**0.001**
NT-proBNP, pg/mL	1183 (267-2791)	1726 (657-3517)	2663 (1225-7495)	5834 (2820-12857)	**0.001**
NT-proBNP (eGFR corrected), pg/mL	1201 (325-2807)	1261 (524-2347)	1643 (667-3548)	2345 (1195-4832)	**0.001**
Cardiac troponin I, µg/L	0.02 (0.02-0.20)	0.02 (0.02-0.11)	0.03 (0.02-0.23)	0.05 (0.02-0.20)	**0.001**
**Medication at discharge**, n (%)					
ACE-inhibitor	263 (51.6)	288 (56.8)	274 (54.5)	190 (40.4)	**0.001**
ARB	103 (20.2)	119 (23.5)	129 (25.6)	112 (23.8)	0.223
Beta-blocker	380 (74.5)	382 (75.3)	401 (79.7)	384 (81.7)	**0.017**
Aldosterone antagonist	50 (9.8)	69 (13.6)	79 (15.7)	72 (15.3)	**0.025**
ARNI	5 (1.0)	4 (0.8)	5 (1.0)	8 (1.7)	0.547
SGLT2-inhibitor	23 (4.5)	24 (4.7)	24 (4.8)	9 (1.9)	0.068
Loop diuretics	129 (25.3)	186 (36.7)	268 (53.3)	371 (78.9)	**0.001**
Statin	357 (70.0)	370 (73.0)	362 (72.0)	286 (60.9)	**0.001**
Digitalis	21 (4.1)	18 (3.6)	27 (5.4)	32 (6.8)	0.089
Amiodarone	10 (2.0)	9 (1.8)	20 (4.0)	16 (3.4)	0.088
ASA	275 (53.9)	280 (55.2)	249 (49.5)	207 (44.0)	**0.002**
P2Y12-inhibitor	172 (33.7)	183 (36.1)	172 (34.2)	111 (23.6)	**0.001**
DOAC	141 (27.6)	165 (32.5)	184 (36.6)	160 (34.0)	**0.021**

Vitamin K antagonist	28	(5.5)	29	(5.7)	42	(8.3)	43	(9.1)	0.055

ACE, angiotensin-converting enzyme; ADHF, acute decompensated heart failure; ARB, angiotensin receptor blocker; ARNI, angiotensin receptor neprilysin inhibitor; ASA, acetylsalicylic acid; CABG, coronary artery bypass grafting; DOAC, directly acting oral anticoagulant; eGFR, estimated glomerular filtration rate; HbA1c, glycated hemoglobin; HDL, high-density lipoprotein; IQR, interquartile range; IVSd, interventricular septum in diastole; LA, left atrial; LDL, low-density lipoprotein; LVEDD, Left ventricular end-diastolic diameter; LVEF, left ventricular ejection fraction; NT-proBNP, amino-terminal prohormone of brain natriuretic peptide; NYHA, New York Heart Association; PCI, percutaneous coronary intervention; TAPSE, tricuspid annular plane systolic excursion; UAC, urea-to-albumin ratio; WBC, white blood cells. Level of significance $p \leq 0.05$. Bold type indicates statistical significance.

Supplemental Table 3 - Baseline characteristics.

	Q1: ACR <2.06g/mg (*n* = 516)		Q2: ACR ≥2.06 – <2.99g/mg (*n* = 515)		Q3: ACR ≥2.99 - <3.85g/mg (*n* = 517)		Q4: ACR ≥3.85g/mg (*n* = 513)		p value
**Age**, median (IQR)	79	(72-85)	78	(69-84)	72	(61-81)	69	(57-79)	**0.001**
**Male sex**, n (%)	355	(68.8)	342	(66.4)	352	(68.1)	282	(55.0)	**0.001**
**Body mass index,** kg/m^2, median (IQR)	23.2	(25.8-21.2)	24.2	(22.0-26.9)	24.1	(22.0-26.8)	23.9	(21.7-26.6)	**0.011**
**SBP**, mmHg, median (IQR)	139	(120-160)	142	(124-161)	143	(127-163)	146	(130-166)	**0.001**
**DBP**, mmHg, median (IQR)	74	(62-88)	77	(70-88)	80	(70-92)	82	(72-93)	**0.001**
**Heart rate**, bpm, median (IQR)	82	(70-96)	79	(66-96)	81	(70-94)	80	(69-95)	0.203
**Medical history**, n (%)									
Coronary artery disease	248	(48.1)	237	(46.0)	214	(41.4)	140	(27.3)	**0.001**
Prior myocardial infarction	143	(27.7)	128	(24.9)	125	(24.2)	89	(17.3)	**0.001**
Prior PCI	164	(31.8)	152	(29.5)	146	(28.2)	108	(21.1)	**0.001**
Prior CABG	71	(13.8)	60	(11.7)	42	(8.1)	30	(5.8)	**0.001**
Prior valvular surgery	25	(4.8)	25	(4.9)	21	(4.1)	20	(3.9)	0.816
Congestive heart failure	244	(47.3)	180	(35.0)	151	(29.2)	113	(22.0)	**0.001**
Prior LVEF available, n (%)	233	(45.2)	193	(37.5)	172	(33.3)	139	(27.1)	**0.001**
Prior LVEF, median (IQR)	50	(42-60)	52	(41-60)	50	(45-60)	50	(45-60)	0.984
Prior LVEF ≥50%	130	(55.8)	107	(55.4)	98	(57.0)	76	(54.7)	0.981
Prior LVEF 41-49%	46	(19.7)	38	(19.7)	41	(23.8)	35	(25.2)	0.487
Prior LVEF ≤40%	27	(11.6)	21	(10.9)	17	(10.0)	10	(7.2)	0.573
Decompensated heart failure within 12 months prior to index	90	(17.4)	57	(11.1)	40	(7.7)	30	(5.8)	**0.001**
Prior ICD	12	(2.3)	11	(2.1)	10	(1.9)	7	(1.4)	0.706
Prior sICD	2	(0.4)	3	(0.6)	1	(0.2)	2	(0.4)	0.799
Prior CRT-D	10	(1.9)	12	(2.3)	5	(1.0)	2	(0.4)	**0.033**
Prior Pacemaker	69	(13.4)	41	(8.0)	44	(8.5)	34	(6.6)	**0.001**
Chronic kidney disease	419	(81.2)	180	(35.0)	46	(8.9)	3	(0.6)	**0.001**
Peripheral artery disease	98	(19.0)	65	(12.6)	39	(7.5)	28	(5.5)	**0.001**
Stroke	89	(17.2)	90	(17.5)	74	(14.3)	65	(12.7)	0.092
Liver cirrhosis	28	(5.4)	13	(2.5)	0	(0.0)	2	(0.4)	**0.001**
Malignancy	116	(22.5)	85	(16.5)	59	(11.4)	51	(9.9)	**0.001**
COPD	86	(16.7)	71	(13.8)	52	(10.1)	38	(7.4)	**0.001**
**Cardiovascular risk factors,** n (%)									
Arterial hypertension	448	(86.8)	429	(83.3)	385	(74.5)	346	(67.4)	**0.001**
Diabetes mellitus	255	(49.4)	221	(42.9)	158	(30.6)	124	(24.2)	**0.001**
Hyperlipidemia	164	(31.8)	165	(32.0)	167	(32.3)	131	(25.5)	0.052
Smoking	181	(35.1)	177	(34.4)	194	(37.5)	206	(40.2)	0.206
Current	64	(12.4)	80	(15.5)	104	(20.1)	143	(27.9)	**0.001**
Former	117	(22.7)	97	(18.8)	90	(17.4)	63	(12.3)	**0.001**
Family history	22	(4.3)	40	(7.8)	66	(12.8)	61	(11.9)	**0.001**
**Comorbidities at index hospitalization**, n (%)									
Acute coronary syndrome									
Unstable angina	9	(1.7)	24	(4.7)	39	(7.5)	24	(4.7)	**0.001**
STEMI	16	(3.1)	43	(8.3)	59	(11.4)	51	(9.9)	**0.001**
NSTEMI	63	(12.2)	76	(14.8)	67	(13.0)	61	(11.9)	0.522
Acute decompensated heart failure	211	(40.9)	127	(24.7)	73	(14.1)	55	(10.7)	**0.001**
Cardiogenic shock	23	(4.5)	10	(1.9)	11	(2.1)	8	(1.6)	0.013
Atrial fibrillation	252	(48.8)	231	(44.9)	201	(38.9)	175	(34.1)	**0.001**
Cardiopulmonary resuscitation	15	(2.9)	10	(1.9)	17	(3.3)	9	(1.8)	0.317
Out-of-hospital	3	(0.6)	5	(1.0)	9	(1.7)	5	(1.0)	0.326
In-hospital	12	(2.3)	5	(1.0)	8	(1.5)	4	(0.8)	0.146
Stroke	50	(9.7)	77	(15.0)	71	(13.7)	100	(19.5)	**0.001**
**Medication on admission**, n (%)									
ACE-inhibitor	193	(37.4)	203	(39.4)	190	(36.8)	152	(29.6)	**0.007**
ARB	133	(25.8)	128	(24.9)	107	(20.7)	87	(17.0)	**0.002**
Beta-blocker	358	(69.4)	327	(63.5)	259	(50.1)	219	(42.7)	**0.001**
Aldosterone antagonist	57	(11.0)	63	(12.2)	44	(8.5)	21	(4.1)	**0.001**
ARNI	6	(1.2)	5	(1.0)	2	(0.4)	3	(0.6)	0.472
SGLT2-inhibitor	8	(1.6)	11	(2.1)	12	(2.3)	11	(2.1)	0.831
Loop diuretics	325	(63.0)	238	(46.2)	122	(23.6)	84	(16.4)	**0.001**
Statin	262	(50.8)	274	(53.2)	214	(41.4)	181	(35.3)	**0.001**
ASA	189	(36.6)	184	(35.7)	179	(34.6)	142	(27.7)	**0.010**
P2Y12-inhibitor	54	(10.5)	63	(12.2)	37	(7.2)	40	(7.8)	**0.017**
DOAC	139	(26.9)	131	(25.4)	115	(22.2)	99	(19.3)	**0.019**
Vitamin K antagonist	54	(10.5)	59	(11.5)	38	(7.4)	26	(5.1)	**0.001**

ACE, angiotensin-converting-enzyme; ACR, albumin-to-creatinine ratio; ARB, angiotensin receptor blocker; ARNI, angiotensin receptor neprilysin inhibitor; ASA, acetylsalicylic acid; CABG, coronary artery bypass grafting; CKD, chronic kidney disease; COPD, chronic obstructive pulmonary disease; CRT-D, cardiac resynchronization therapy with defibrillator; DBP, diastolic blood pressure; DOAC, directly acting oral anticoagulant; IQR, interquartile range; PCI, percutaneous coronary intervention; (N)STEMI, non-ST-segment elevation myocardial infarction; SBP, systolic blood pressure; SGLT2, sodium glucose linked transporter 2; (s) ICD, (subcutaneous) implantable cardioverter defibrillator. Level of significance p≤0.05. Bold type indicates statistical significance.

Supplemental Table 4 - Heart failure-related and procedural data.

	Q1: ACR <2.06g/mg (*n* = 516)	Q2: ACR ≥2.06 – <2.99g/mg (*n* = 515)	Q3: ACR ≥2.99 - <3.85g/mg (*n* = 517)	Q4: ACR ≥3.85g/mg (*n* = 513)	p value
**Heart failure etiology**, n (%)					
Ischemic cardiomyopathy	297 (57.6)	325 (63.1)	319 (61.7)	250 (48.7)	**0.001**
Non-ischemic cardiomyopathy	38 (7.4)	36 (7.0)	30 (5.8)	43 (8.4)	0.449
Hypertensive cardiomyopathy	40 (7.8)	38 (7.4)	33 (6.4)	53 (10.3)	0.113
Congenital heart disease	1 (0.2)	1 (0.2)	1 (0.2)	1 (0.2)	1.000
Valvular heart disease	29 (5.6)	23 (4.5)	18 (3.5)	23 (4.5)	0.432
Tachycardia associated	24 (4.7)	26 (5.0)	33 (6.4)	37 (7.2)	0.266
Tachymyopathy	7 (1.4)	7 (1.4)	8 (1.5)	14 (2.7)	0.271
Pacemaker-induced cardiomyopathy	8 (1.6)	2 (0.4)	6 (1.2)	3 (0.6)	0.187
Unknown	79 (15.3)	64 (12.4)	77 (14.9)	103 (20.1)	**0.008**
**NYHA functional class,** n (%)					
I/II	291 (56.4)	351 (68.1)	472 (82.6)	430 (83.8)	
III	143 (27.7)	114 (22.1)	68 (13.2)	60 (11.7)	**0.001**
IV	82 (15.9)	50 (9.7)	22 (4.3)	23 (4.5)	
**Echocardiographic data**					
LVEF, %, median (IQR)	45 (45-47)	45 (45-47)	45 (45-47)	45 (45-47)	0.273
IVSd, median (IQR)	12 (11-13)	12 (11-13)	12 (11-13)	11 (10-13)	**0.043**
LVEDD, mm, median (IQR)	50 (45-55)	48 (44-53)	49 (44-54)	49 (44-53)	**0.002**
TAPSE, mm, median (IQR)	19 (16-22)	20 (17-23)	20 (17-23)	20 (18-23)	**0.001**
LA diameter, mm, median (IQR)	44 (39-49)	42 (38-48)	41 (36-46)	39 (34-45)	**0.001**
LA surface, cm^2, median (IQR)	24 (20-28)	22 (18-26)	21 (16-25)	20 (16-23)	**0.001**
Moderate-severe aortic stenosis, n (%)	71 (13.8)	57 (11.1)	38 (7.4)	34 (6.6)	**0.001**
Moderate-severe aortic regurgitation, n (%)	28 (5.4)	22 (4.3)	16 (3.1)	13 (2.5)	0.074
Moderate-severe mitral regurgitation, n (%)	91 (17.6)	59 (11.5)	53 (10.3)	47 (9.2)	**0.001**
Moderate-severe tricuspid regurgitation, n (%)	130 (25.2)	85 (16.5)	63 (12.2)	43 (8.4)	**0.001**
**Coronary angiography,** n (%)	142 (27.5)	232 (45.0)	262 (50.7)	233 (45.4)	**0.001**
No evidence of coronary artery disease	26 (18.3)	44 (19.0)	41 (15.6)	61 (26.2)	
1-vessel disease	15 (10.6)	40 (17.2)	54 (20.6)	52 (22.3)	**0.002**
2-vessel disease	26 (18.3)	50 (21.6)	60 (22.9)	48 (20.6)	
3-vessel disease	75 (52.8)	98 (42.2)	107 (40.8)	72 (30.9)	
Prior CABG	12 (8.5)	16 (6.9)	13 (5.0)	8 (3.4)	0.161
Chronic total occlusion	19 (13.4)	28 (12.1)	37 (14.1)	24 (10.3)	0.614
PCI, n (%)	65 (45.8)	122 (52.6)	153 (58.4)	126 (54.1)	0.110
Sent to CABG, n (%)	16 (11.3)	27 (11.6)	16 (6.1)	12 (5.2)	**0.020**
**Baseline laboratory values**, median (IQR)					
Potassium, mmol/L	4.0 (3.6-4.3)	3.9 (3.6-4.2)	3.9 (3.6-4.1)	3.8 (3.6-4.1)	**0.001**
Sodium, mmol/L	139 (137-142)	139 (137-141)	139 (137-141)	139 (138-141)	0.353
Albumin, g/dL	27.6 (23.3-31.8)	31.6 (28.1-35.0)	33.8 (31.1-36.6)	35.3 (32.2-38.2)	**0.001**
Urea, mg/dL	75.0 (52.2-114.3)	44.2 (35.1-57.6)	36.0 (29.3-44.0)	28.6 (23.2-35.5)	**0.001**
Creatinine, mg/dL	2.02 (1.60-2.92)	1.22 (1.07-1.38)	1.00 (0.90-1.08)	0.77 (0.67-0.86)	**0.001**
eGFR, mL/min/1.73 m^2	31 (20-41)	56 (48-67)	74 (64-85)	96 (85-108)	**0.001**
Hemoglobin, g/dL	10.3 (8.9-11.8)	12.2 (10.4-13.7)	13.3 (11.7-14.5)	13.4 (12.0-14.7)	**0.001**
Iron, µg/dL	7.3 (5.2-11.2)	7.4 (5.0-11.9)	8.1 (5.0-12.3)	8.5 (5.8-14.5)	0.241
Ferritin, ng/mL	208 (75-457)	117 (42-241)	125 (56-341)	131 (54-278)	**0.003**
Transferrin, mg/dL	1.8 (1.5-2.3)	2.2 (1.8-2.6)	2.0 (1.6-2.6)	2.1 (1.7-2.5)	**0.001**
Transferrin saturation, %	19 (14-29)	18 (13-25)	21 (15-26)	22 (15-32)	0.400
WBC count, x 10^9/L	8.9 (6.7-11.1)	8.0 (6.3-9.9)	8.1 (6.4-10.1)	8.2 (6.5-9.7)	**0.001**
Platelet count, x 10^9/L	222 (167-286)	225 (175-295)	228 (180-277)	230 (192-283)	0.062
HbA1c, %	6.1 (5.6-7.4)	6.1 (5.6-7.0)	5.8 (5.5-6.7)	5.7 (5.3-6.2)	**0.001**
Total cholesterol, mg/dL	138 (111-166)	149 (122-181)	161 (130-194)	172 (142-198)	**0.001**
LDL-cholesterol, mg/dL	81 (62-106)	91 (67-118)	102 (78-132)	110 (82-133)	**0.001**
HDL-cholesterol, mg/dL	39 (31-46)	42 (35-51)	42 (34-51)	44 (36-58)	**0.001**
C-reactive protein, mg/L	34 (11-79)	15 (5-45)	9 (3-32)	7 (3-18)	**0.001**
NT-proBNP, pg/mL	8133 (3719-17711)	2571 (1207-5103)	1703 (538-3157)	1419 (306-3266)	**0.001**
NT-proBNP (eGFR corrected), pg/mL	2580 (1301-5323)	1454 (633-2677)	1322 (530-2885)	1298 (414-2942)	**0.001**
Cardiac troponin I, µg/L	0.05 (0.02-0.17)	0.03 (0.02-0.18)	0.02 (0.02-0.23)	0.02 (0.02-0.16)	**0.001**
**Medication at discharge**, n (%)					
ACE-inhibitor	192 (40.6)	262 (52.2)	285 (56.4)	276 (54.1)	**0.001**
ARB	116 (24.5)	136 (27.1)	113 (22.4)	98 (19.2)	**0.024**
Beta-blocker	378 (79.9)	412 (82.1)	385 (76.2)	372 (72.9)	**0.003**
Aldosterone antagonist	58 (12.3)	90 (17.9)	68 (13.5)	54 (10.6)	**0.005**
ARNI	7 (1.5)	5 (1.0)	3 (0.6)	7 (1.4)	0.531
SGLT2-inhibitor	12 (2.5)	18 (3.6)	25 (5.0)	25 (4.9)	0.164
Loop diuretics	364 (77.0)	296 (59.0)	166 (32.9)	128 (25.1)	**0.001**
Statin	284 (60.0)	367 (73.1)	364 (72.1)	360 (70.6)	**0.001**
Digitalis	23 (4.9)	31 (6.2)	22 (4.4)	22 (4.3)	0.487
Amiodarone	23 (4.9)	11 (2.2)	16 (3.2)	5 (1.0)	**0.002**
ASA	212 (44.8)	247 (49.2)	285 (56.4)	267 (52.4)	**0.003**
P2Y12-inhibitor	110 (23.3)	171 (34.1)	186 (36.8)	171 (33.5)	**0.001**

DOAC	148	(31.3)	186	(37.1)	168	(33.3)	148	(29.0)	**0.047**
Vitamin K antagonist	44	(9.3)	42	(8.4)	30	(5.9)	26	(5.1)	**0.032**

ACE, angiotensin-converting enzyme; ACR, albumin-to-creatinine ratio; ADHF, acute decompensated heart failure; ARB, angiotensin receptor blocker; ARNI, angiotensin receptor neprilysin inhibitor; ASA, acetylsalicylic acid; CABG, coronary artery bypass grafting; DOAC, directly acting oral anticoagulant; eGFR, estimated glomerular filtration rate; HbA1c, glycated hemoglobin; HDL, high-density lipoprotein; IQR, interquartile range; IVSd, interventricular septum in diastole; LA, left atrial; LDL, low-density lipoprotein; LVEDD, Left ventricular end-diastolic diameter; LVEF, left ventricular ejection fraction; NT-proBNP, amino-terminal prohormone of brain natriuretic peptide; NYHA, New York Heart Association; PCI, percutaneous coronary intervention; TAPSE, tricuspid annular plane systolic excursion; WBC, white blood cells. Level of significance $p \leq 0.05$. Bold type indicates statistical significance.

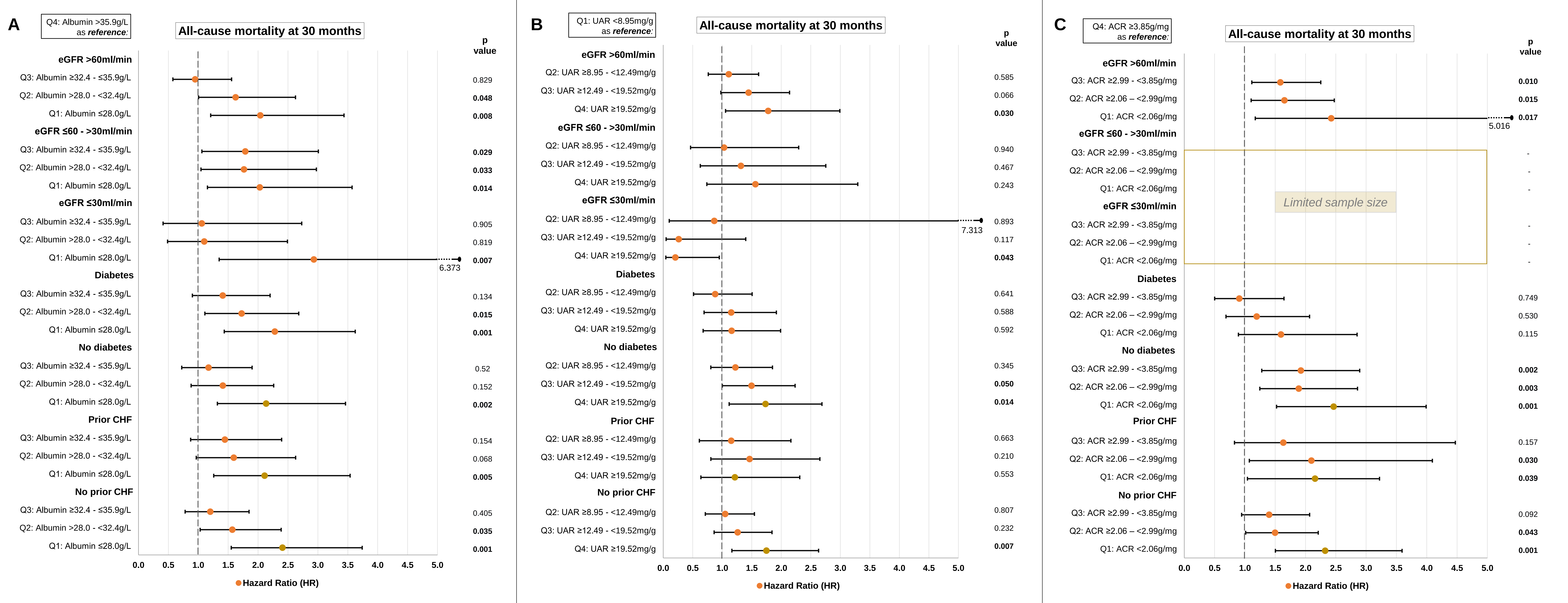

A
Q4: Albumin >35.9g/L as reference:
All-cause mortality at 30 months
p value
eGFR >60ml/min
Q3: Albumin ≥32.4 - ≤35.9g/L 0.829
Q2: Albumin >28.0 - <32.4g/L 0.048
Q1: Albumin ≤28.0g/L 0.008
eGFR ≤60 - >30ml/min
Q3: Albumin ≥32.4 - ≤35.9g/L 0.029
Q2: Albumin >28.0 - <32.4g/L 0.033
Q1: Albumin ≤28.0g/L 0.014
eGFR ≤30ml/min
Q3: Albumin ≥32.4 - ≤35.9g/L 0.905
Q2: Albumin >28.0 - <32.4g/L 0.819
Q1: Albumin ≤28.0g/L 0.007
6.373
Diabetes
Q3: Albumin ≥32.4 - ≤35.9g/L 0.134
Q2: Albumin >28.0 - <32.4g/L 0.015
Q1: Albumin ≤28.0g/L 0.001
No diabetes
Q3: Albumin ≥32.4 - ≤35.9g/L 0.52
Q2: Albumin >28.0 - <32.4g/L 0.152
Q1: Albumin ≤28.0g/L 0.002
Prior CHF
Q3: Albumin ≥32.4 - ≤35.9g/L 0.154
Q2: Albumin >28.0 - <32.4g/L 0.068
Q1: Albumin ≤28.0g/L 0.005
No prior CHF
Q3: Albumin ≥32.4 - ≤35.9g/L 0.405
Q2: Albumin >28.0 - <32.4g/L 0.035
Q1: Albumin ≤28.0g/L 0.001
0.0 0.5 1.0 1.5 2.0 2.5 3.0 3.5 4.0 4.5 5.0
Hazard Ratio (HR)
B
Q1: UAR <8.95mg/g as reference:
All-cause mortality at 30 months
p value
eGFR >60ml/min
Q2: UAR ≥8.95 - <12.49mg/g 0.585
Q3: UAR ≥12.49 - <19.52mg/g 0.066
Q4: UAR ≥19.52mg/g 0.030
eGFR ≤60 - >30ml/min
Q2: UAR ≥8.95 - <12.49mg/g 0.940
Q3: UAR ≥12.49 - <19.52mg/g 0.467
Q4: UAR ≥19.52mg/g 0.243
eGFR ≤30ml/min
Q2: UAR ≥8.95 - <12.49mg/g 0.893
7.313
Q3: UAR ≥12.49 - <19.52mg/g 0.117
Q4: UAR ≥19.52mg/g 0.043
Diabetes
Q2: UAR ≥8.95 - <12.49mg/g 0.641
Q3: UAR ≥12.49 - <19.52mg/g 0.588
Q4: UAR ≥19.52mg/g 0.592
No diabetes
Q2: UAR ≥8.95 - <12.49mg/g 0.345
Q3: UAR ≥12.49 - <19.52mg/g 0.050
Q4: UAR ≥19.52mg/g 0.014
Prior CHF
Q2: UAR ≥8.95 - <12.49mg/g 0.663
Q3: UAR ≥12.49 - <19.52mg/g 0.210
Q4: UAR ≥19.52mg/g 0.553
No prior CHF
Q2: UAR ≥8.95 - <12.49mg/g 0.807
Q3: UAR ≥12.49 - <19.52mg/g 0.232
Q4: UAR ≥19.52mg/g 0.007
0.0 0.5 1.0 1.5 2.0 2.5 3.0 3.5 4.0 4.5 5.0
Hazard Ratio (HR)
C
Q4: ACR ≥3.85g/mg as reference:
All-cause mortality at 30 months
p value
eGFR >60ml/min
Q3: ACR ≥2.99 - <3.85g/mg 0.010
Q2: ACR ≥2.06 – <2.99g/mg 0.015
Q1: ACR <2.06g/mg 0.017
5.016
eGFR ≤60 - >30ml/min
Q3: ACR ≥2.99 - <3.85g/mg -
Q2: ACR ≥2.06 – <2.99g/mg -
Q1: ACR <2.06g/mg -
Limited sample size
eGFR ≤30ml/min
Q3: ACR ≥2.99 - <3.85g/mg -
Q2: ACR ≥2.06 – <2.99g/mg -
Q1: ACR <2.06g/mg -
Diabetes
Q3: ACR ≥2.99 - <3.85g/mg 0.749
Q2: ACR ≥2.06 – <2.99g/mg 0.530
Q1: ACR <2.06g/mg 0.115
No diabetes
Q3: ACR ≥2.99 - <3.85g/mg 0.002
Q2: ACR ≥2.06 – <2.99g/mg 0.003
Q1: ACR <2.06g/mg 0.001
Prior CHF
Q3: ACR ≥2.99 - <3.85g/mg 0.157
Q2: ACR ≥2.06 – <2.99g/mg 0.030
Q1: ACR <2.06g/mg 0.039
No prior CHF
Q3: ACR ≥2.99 - <3.85g/mg 0.092
Q2: ACR ≥2.06 – <2.99g/mg 0.043
Q1: ACR <2.06g/mg 0.001
0.0 0.5 1.0 1.5 2.0 2.5 3.0 3.5 4.0 4.5 5.0
Hazard Ratio (HR)

Supplemental Table 5 - Univariable Cox regression analyses regarding 30-month all-cause mortality and HF-related rehospitalization in the entire study cohort (n = 2061).

	All-cause mortality		
**Variables**	**HR**	**95% CI**	**p value**
Age (years)	1.054	1.046-1.062	**0.001**
Male sex	0.927	0.789-1.088	0.351
BMI (kg/m^2)	0.934	0.917-0.952	**0.001**
Chronic kidney disease	2.478	2.133-2.894	**0.001**
Malignancy	3.126	2.632-3.712	**0.001**
Prior congestive heart failure	1.548	1.324-1.811	**0.001**
Prior CAD	1.081	0.924-1.264	0.331
Diabetes mellitus	1.279	1.094-1.496	**0.002**
Acute decompensated heart failure	2.298	1.955-2.700	**0.001**
Ischemic cardiomyopathy	0.773	0.662-0.903	**0.001**
Right ventricular dysfunction*	1.903	1.602-2.259	**0.001**
Hemoglobin (g/dL)	0.770	0.744-0.796	**0.001**
C-reactive protein (mg/L)	1.005	1.004-1.006	**0.001**

BMI, body mass index; CAD, coronary artery disease; CI, confidence interval; HF, heart failure; HR, hazard ratio. Level of significance p<0,05. Bold type indicates statistical significance. *Right ventricular dysfunction was defined by a Tricuspid Annular Plane Systolic Excursion (TAPSE) <17mm.
